# Identification of prognostic long noncoding RNAs associated with spontaneous regression of neuroblastoma

**DOI:** 10.1002/cam4.3022

**Published:** 2020-03-26

**Authors:** Xinyao Meng, Erhu Fang, Xiang Zhao, Jiexiong Feng

**Affiliations:** ^1^ Department of Pediatric Surgery Tongji Hospital Tongji Medical College Huazhong University of Science and Technology Wuhan China

**Keywords:** long noncoding RNA, neuroblastoma, prognostic, spontaneous regression

## Abstract

**Background:**

The association between long noncoding RNAs (lncRNAs) and spontaneous regression of neuroblastoma (NB) has rarely been investigated and remains unknown.

**Objective:**

To identify prognostic lncRNAs involved in the spontaneous regression of NB.

**Methods:**

Differential expression analyses were performed between those samples with an outcome of death in stage 4 NB group and those samples with an outcome of survival in stage 4S NB group in two independent public datasets, respectively. Univariate Cox proportional hazard regression survival analysis was performed in each of the entire cohort to identify those lncRNAs significantly associated with overall survival (OS). Those lncRNAs independently associated with OS were then identified by multivariate Cox survival analysis and used to construct an lncRNA risk score.

**Results:**

A total of 20 differentially expressed and survival‐related lncRNAs were identified sharing between the two independent cohorts. The expression of each of these 20 lncRNAs was significantly correlated with the expression of NTRK1, which is a well‐known factor involved in NB spontaneous regression. Four lncRNAs (LNC00839, FIRRE, LOC283177, and LOC101928100) were identified to be significantly associated with survival independent with each other and a four‐lncRNA signature risk score was constructed. Patients with high lncRNA signature risk score had a significantly poorer OS and event‐free survival than those with low lncRNA signature risk score. The four‐lncRNA signature has a good performance in predicting survival independent with MYCN amplification (nonamplified vs amplified), age status (<18 months vs ≥18 months), risk status (low risk vs high risk), and International Neuroblastoma Staging System (INSS) stage (INSS 1/2/3/4S vs INSS 4).

**Conclusions:**

We identified 20 survival‐related lncRNAs that might be associated with the spontaneous regression of NB and developed a four‐lncRNA signature risk score. The four‐lncRNA signature is an independent prognostic factor for survival of NB patients.

## Graphical abstract

We identified 20 survival‐related long noncoding RNAs (lncRNAs) that might be associated with the spontaneous regression of neuroblastoma (NB) and developed a four‐lncRNA signature risk score. The four‐lncRNA signature is an independent prognostic factor of survival in NB.

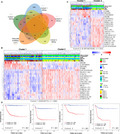

## 1 INTRODUCTION

Spontaneous regression has been observed in different type of cancers including hepatocellular carcinoma, malignant melanoma, thoracic malignancies, lung cancer, and neuroblastoma (NB).^1^, ^2^, ^3^, ^4^, ^5^ However, NB is generally considered the malignancy, in which this phenomenon is most prevalent.^2^ The spontaneous regression of NB has been well documented by mass screening programs undertaken in Japan,^6^, ^7^ North America,^8^ and Europe,^9^ and it is most evident in infants with stage 4S disease.^2^, ^10^, ^11^, ^12^ In 1971, Evans and D'Angio identified a specific group of metastatic NB spread in infants under 1 year of age that they called stage IVS.^13^, ^14^ Infants with stage IVS NB generally had a small primary tumor, but with metastatic disease in the liver, skin, and bone marrow, or any combination of these. Unlike other metastatic malignancies, this group of NB patients generally had a surprisingly good prognosis and some even underwent spontaneous regression without tumor‐specific therapy.^13^, ^14^ The definition of stage IVS was refined by the International Neuroblastoma Staging System (INSS) as stage 4S and by the International Neuroblastoma Risk Group Staging System (INRGSS) as stage metastatic special (MS), and the children up to 18 months of age can be considered to have stage MS.^15^, ^16^ In fact, spontaneous regression of NB is not restricted to stage 4S; it can be seen in infants with any stage of NB if they have biologically favorable tumors.^2^, ^14^ However, it is difficult to know for certain which NB will regress based on age and stage alone. Since spontaneous regression of NB is most evident in infants with stage 4S disease, investigators have focused on stage 4S tumors as a surrogate to investigate the mechanisms underlining spontaneous regression.^10^, ^11^, ^12^

Recent years, long noncoding RNAs (lncRNAs) have been proved to play important roles in various types of cancers and are emerging as attractive candidates for noninvasive biomarkers and therapeutic targets for the treatment of cancer.^17^, ^18^ Increasing evidence also revealed that lncRNAs are involved in NB either as oncogene or as tumor suppressor.^19^, ^20^, ^21^, ^22^, ^23^, ^24^ However, the association between lncRNAs and spontaneous regression of NB has rarely been investigated partially due to the lack of NB tissue samples, which underwent spontaneous regression. In this study, we performed differential expression analyses between those cases with an outcome of death from stage 4 NB group and those cases with an outcome of survival from stage 4S NB group in two independent cohorts, respectively. Excluding the dead cases in stage 4S would make it better for stage 4S cases to serve as surrogates to NBs underwent spontaneous regression. Univariate Cox proportional hazard regression survival analysis was performed to identify those lncRNAs significantly associated with overall survival (OS). Finally, we identified 20 differentially expressed and survival‐related lncRNAs. We also found that the expression of each of these 20 lncRNAs was statistically correlated with the expression of NTRK1, which is a well‐known factor involved in NB spontaneous regression.^25^, ^26^ This result indicates the involvement of these 20 lncRNAs in NB spontaneous regression. We also build a four‐lncRNA signature that has a good performance in predicting OS and event‐free survival (EFS) in NB patients.

## 2 MATERIALS AND METHODS

### 2.1 NB patient datasets

NB patient datasets and corresponding clinical information were collected from the publicly available Gene Expression Omnibus (GEO) database and ArrayExpress database. After filtering out datasets without enough survival information, two microarray datasets were selected: GSE49710 (n = 498) from the GEO datasets and E‐MTAB‐8248 (n = 223) from the ArrayExpress database. Both of the two datasets are Agilent microarrays performed on platform GPL16876 (Agilent‐020382 Human Custom Microarray 44k). Microarray dataset GSE49710 was used as the discovery cohort and termed as cohort 1. Microarray dataset E‐MTAB‐8248 was used as the validation cohort and termed as cohort 2. The clinical characteristics of the two cohorts were shown in Table 1. The genomic alteration (mutation and copy number alteration) of the identified lncRNAs were analyzed on the open platform of cBio Cancer Genomics Portal (cBioportal) (http://www.cbioportal.org/),^27^ which provides mutation data for 755 cases of NB and copy number alteration data for 59 cases of NB.

**TABLE 1:** The clinical characteristics of cohort 1 and cohort 2

	No. of cohort 1 (%)	No. of cohort 2 (%)	*P* value
Age			<.001
< 18 mo	300 (60.2)	103 (46.2)	
≥ 18 mo	198 (39.8)	120 (53.8)	
Sex			—
Male	287 (57.6)	—	
Female	211 (42.4)	—	
MYCN status			.519
Nonamplified	401 (80.5)	176 (78.9)	
Amplified	92 (18.5)	46 (20.6)	
Risk			—
Low	322 (64.7)	—	
High	176 (35.3)	—	
INSS stage			.013
1	121 (24.3)	29 (13.0)	
2	78 (15.7)	39 (17.5)	
3	63 (12.7)	36 (16.1)	
4	183 (36.7)	89 (39.9)	
4S	53 (10.6)	30 (13.5)	
Death			.488
Yes	105 (21.1)	42 (18.8)	
No	393 (78.9)	181 (81.2)	
Progression			.418
Yes	183 (36.7)	89 (39.9)	
No	315 (63.3)	134 (60.1)	

### 2.2 Microarray processing and lncRNA mining

The processed data of the two NB datasets were downloaded from their database. The Agilent microarray probes IDs were first annotated using the platform GPL16876. Then, in order to renew the annotation and identify more newly validated lncRNAs, the probes IDs were reannotated according to their corresponding Genebank Accession number by BRB‐ArrayTools software (version 4.6.0), which utilizes the R (version 3.5.1) and Bioconductor package.^28^ Differential expression analyses were performed using Significance Analysis of Microarrays (SAM) method by the BRB‐ArrayTools software. SAM analysis was performed using stringent statistics variables with a false discovery rate of <0.1%, and permutation of 1000. Only those lncRNAs matched to GENCODE annotation of lncRNAs (release 31, GRCh38.p12) were selected. When multiple probes mapped to the same lncRNA, the mean of the probe intensities will be used. Heatmap and clustering analysis was performed by TMeV software (TIGR MultiExperiment Viewer, version 4.9.0). Venn diagram was made by the online available Venn diagram viewer jvenn.^29^

### 2.3 Statistical analysis

To identify lncRNAs predictive of OS, a univariate Cox proportional hazards regression analysis was performed to evaluate the relationship between the continuous expression level of each lncRNA and patient OS. Only those lncRNAs correlated with OS with *P* values of ≤.001 were considered statistically significant. Multivariable Cox proportional hazard regression analysis was performed to determine the prognostic independence of those selected lncRNAs. Those lncRNAs with a *P* value of <.005 by multivariable Cox regression model were considered to be associated with survival independently. Only those lncRNAs independently associated with OS were used to construct the lncRNA risk score. The risk score was computed as follows:

$$\text{Risk score}=\sum_{i=1}^{N}\left(E_{i}\times W_{i}\right).$$

where *N* is the number of prognostic lncRNAs, *E_i_* is the expression value of lncRNAi, and *W_i_* is the multivariate coefficient for lncRNAi.

The time‐dependent receiver operating characteristic (ROC) curve and area under curve (AUC) analyses were used to evaluate the sensitivity and specificity of the lncRNAs or lncRNA risk score for survival prediction. The optimal cutoff values for those lncRNAs or lncRNA signature risk score were determined by Youden's index (sensitivity + specificity − 1). The value which has the highest Youden's index was chosen as the optimal cutoff value. The cohort was divided into two groups according to the optimal cutoff value. Kaplan‐Meier survival analyses were performed to test the survival probability in different groups for each cohort, and statistical significance was assessed using the two‐sided log‐rank test. Survival analysis and ROC curve analyses were performed using SPSS 18 and STATA 15 software. The time‐dependent receiver ROC curve and AUC analyses were constructed by R (version 3.5.1) software. The differences of categorical data between groups were evaluated by the chi‐square test, and a *P* value of ≤.05 were considered statistically significant. All statistical tests were two‐sided.

### 2.4 Function prediction of the lncRNA signature

LncRNAs have no protein‐coding capacity; they usually function by regulating the expression of protein‐coding genes. We applied a guilty‐by‐association strategy to investigate the potential biological function of the lncRNA signature. Pearson correlation analyses were performed to identify those genes correlated with each of the lncRNA. Only those genes with a correlation threshold ≥0.3 were extracted. Gene functional annotations (KEGG pathways and GO biological process) were carried out using the online platform Metascape (http://metascape.org/).^30^ Functional annotation with a *P* value of <.01 and an enrichment score of >1.5 were considered statistically significant.

## 3 RESULTS

### 3.1 Identification of differentially expressed and survival‐related lncRNAs

Screen analyses were first performed separately on cohort 1 (GSE49710, n = 498) and cohort 2 (E‐MTAB‐8248, n = 223). Cohort 1 contains 183 stage 4 NB samples and 54 stage 4S NB samples. Cohort 2 contains 89 stage 4 NB samples and 30 stage 4S NB samples. Differential expression analysis was performed between those samples with an outcome of death in stage 4 group (n = 82 in cohort 1 and n = 29 in cohort 2) and those samples with an outcome of survival in stage 4S group (n = 49 in cohort 1 and n = 29 in cohort 2) in each of the cohort, respectively. Univariate survival analysis was performed in each of the entire cohort to identify those genes significantly (*P* < .001) associated with OS. 241 differentially expressed and OS‐related lncRNAs were identified in cohort 1, and 22 differentially expressed and OS‐related lncRNAs were identified in cohort 2.

**FIGURE 1:**
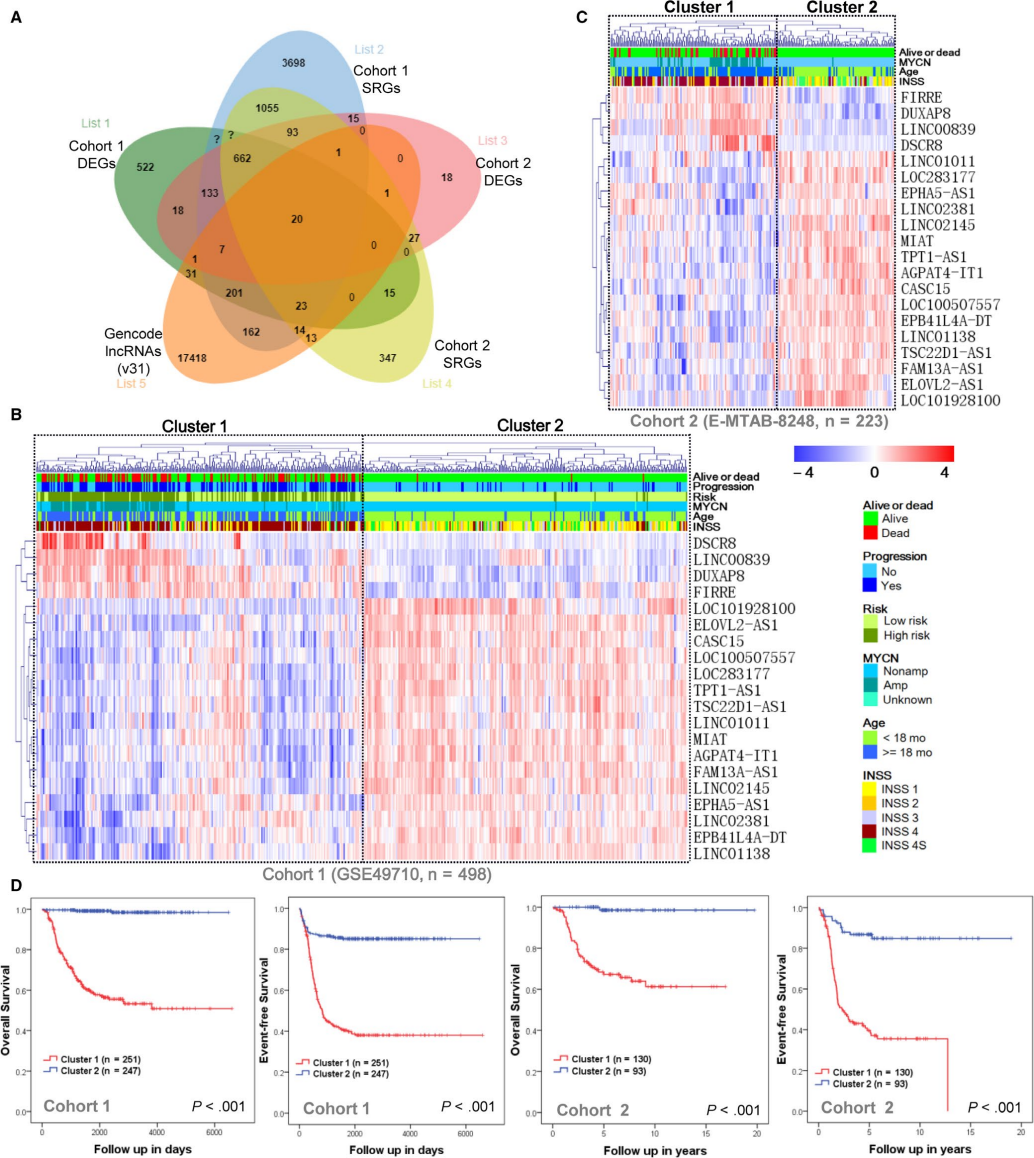
Identification of differentially expressed and survival‐related lncRNAs. A, Venn diagram shows overlapping lncRNAs shared between DEGs in cohort 1, SRGs in cohort 1, DEGs in cohort 2, SRGs in cohort 2, and GENCODE lncRNAs (release 31, GRCh38.p12). B, The heatmap shows the expression values of the identified 20 lncRNAs in cohort 1. Each column indicates a patient noted according to their clinical information related to survival outcome, progression status, risk status, age, MYCN status, and INSS stage. Each row represents lncRNAs ordered by complete linkage hierarchical clustering. The expression value of each lncRNA was z‐normalized and is shown with a blue‐red gradient color scale. The hierarchical clustering identified two distinct clusters, as demonstrated by boxes. C, The heatmap shows the expression values of the identified 20 lncRNAs in cohort 2. D, Kaplan‐Meier plot of OS and EFS for cluster 1 and cluster 2 in each cohort. The *P* values were obtained using a Mantel log‐rank test (two‐sided). DEGs, differentially expressed genes; EFS, event‐free survival; INSS, International Neuroblastoma Staging System; lncRNA, long noncoding RNA; OS, overall survival; SRGs, survival‐related genes

Overlap analysis finally identified 20 lncRNAs shared between cohort 1 and cohort 2 (Figure 1A and Table 2). Four (LINC00839, FIRRE, DUXAP8, and DSCR8) of the 20 identified lncRNAs are upregulated in stage 4 NB samples, and other 16 lncRNAs (LINC02381, EPHA5‐AS1, LOC101928100, ELOVL2‐AS1, EPB41L4A‐DT, LINC01138, LINC01011, LOC100507557, CASC15, AGPAT4‐IT1, MIAT, TPT1‐AS1, LOC283177, TSC22D1‐AS1, LINC02145, and FAM13A‐AS1) are upregulated in stage 4S NB samples. The hierarchical clustering analysis revealed that there were two distinct patients clusters identified in cohort 1 (Figure 1B) and cohort 2 (Figure 1C), respectively. Most of the patients with a bad survival outcome belong to cluster 1 in each of the two cohorts, respectively. While patients from cluster 2 had a good survival outcome, with only three patients died in cohort 1 and one patient died in cohort 2 during the follow‐up period. Kaplan‐Meier plots revealed that patients from cluster 1 had poorer OS and EFS than patients from cluster 2 in each of the two cohorts, respectively (Figure 1D). In cohort 1, the OS rates at 10 years were 59.4% in cluster 1 compared with 98.8% in cluster 2, while the EFS rates at 10 years were 41.4% in cluster 1 compared with 85.4% in cluster 2.

**TABLE 2:** The identified 20 lncRNAs shared between cohort 1 and cohort 2

Ensembl id	Accession	Gene symbol	Chromosomal location	Fold‐change	
				Cohort 1	Cohort 2
ENSG00000185904	NM_032770	LINC00839	10q11.21	7	5.89
ENSG00000206195	NR_122113	DUXAP8	22q11.1	5.68	6.48
ENSG00000213468	NR_026975	FIRRE	Xq26.2	4.76	6.22
ENSG00000198054	NM_032589	DSCR8	21q22.13	3.64	8.39
ENSG00000244041	NM_207495	LINC01011	6p25.2	0.59	0.63
ENSG00000278156	NR_038381	TSC22D1‐AS1	13q14.11	0.56	0.56
ENSG00000170919	NR_024458	TPT1‐AS1	13q14.13	0.48	0.5
ENSG00000250742	NR_026656	LINC02381	12q13.13	0.47	0.38
ENSG00000279355	NM_024929	AGPAT4‐IT1	6q26	0.43	0.49
ENSG00000235652	NR_038244	LOC100507557	6q24.3	0.43	0.36
ENSG00000272168	NR_015410	CASC15	6p22.3	0.39	0.48
ENSG00000278921	NR_027706	EPB41L4A‐DT	5q22.2	0.39	0.45
ENSG00000255545	NR_033852	LOC283177	11q25	0.38	0.47
ENSG00000250490	NM_001001702	LINC02145	5p15.31	0.35	0.43
ENSG00000230314	NR_038962	ELOVL2‐AS1	6p24.2	0.34	0.39
ENSG00000225783	NR_003491	MIAT	22q12.1	0.34	0.45
ENSG00000248019	NR_002806	FAM13A‐AS1	4q22.1	0.33	0.45
ENSG00000250846	NR_034138	EPHA5‐AS1	4q13.2	0.32	0.25
ENSG00000274020	NM_207400	LINC01138	1q21.2	0.26	0.31
ENSG00000245648	NR_120430	LOC101928100	12p13.2	0.14	0.28

### 3.2 Identification of lncRNAs independently associated with survival

**FIGURE 2:**
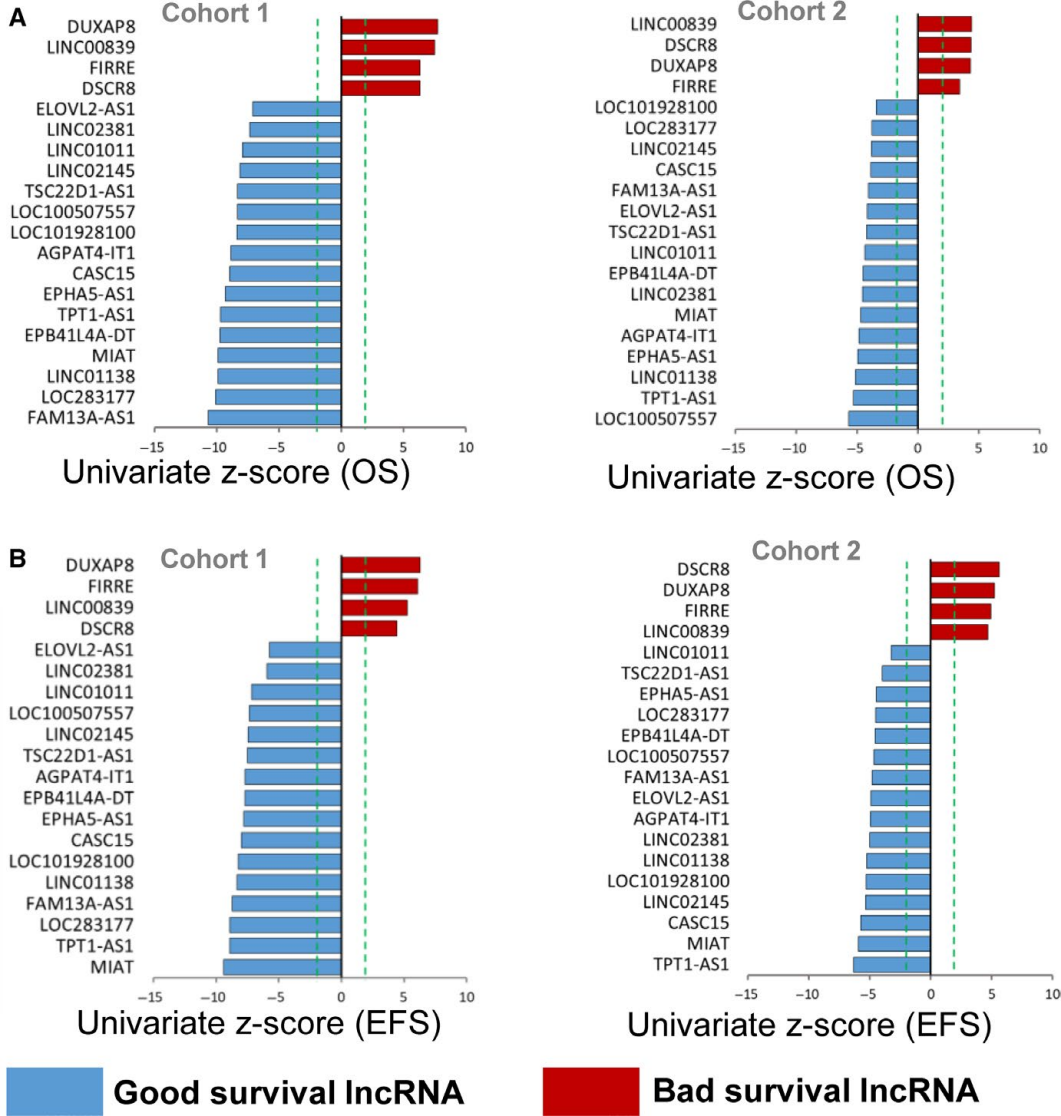
Univariate Cox survival analysis of the identified 20 lncRNAs. A, Bar graphs shows 20 prognostic lncRNAs ordered by their univariate *z*‐score for OS in cohort 1 and cohort 2, respectively. B, Bar graphs shows 20 prognostic lncRNAs ordered by their univariate *z*‐score for EFS in cohort 1 and cohort 2, respectively. The dashed line (colored in green) represents an absolute univariate *z*‐score value of ±1.96. Red bars represent “bad survival lncRNAs,” of which high expression is associated with bad survival. Blue bars represent “good survival lncRNAs,” of which high expression is associated with good survival. EFS, event‐free survival; lncRNA, long noncoding RNA; OS, overall survival

Univariate Cox proportional hazards model survival analysis revealed that the expression level of each of the 20 lncRNAs was significantly (*P* ≤ .001) associated with not only OS (Figure 2A), but also EFS (Figure 2B) in both of the two cohorts (Table S1). The four stage 4 upregulated lncRNAs, which have positive coefficients and of which the high expression is associated with bad survival, were defined as “bad survival lncRNAs.” The remaining 16 stage 4S upregulated lncRNAs, which have negative coefficients and of which the high expression is associated with good survival, were defined as “good survival lncRNAs.”

**FIGURE 3:**
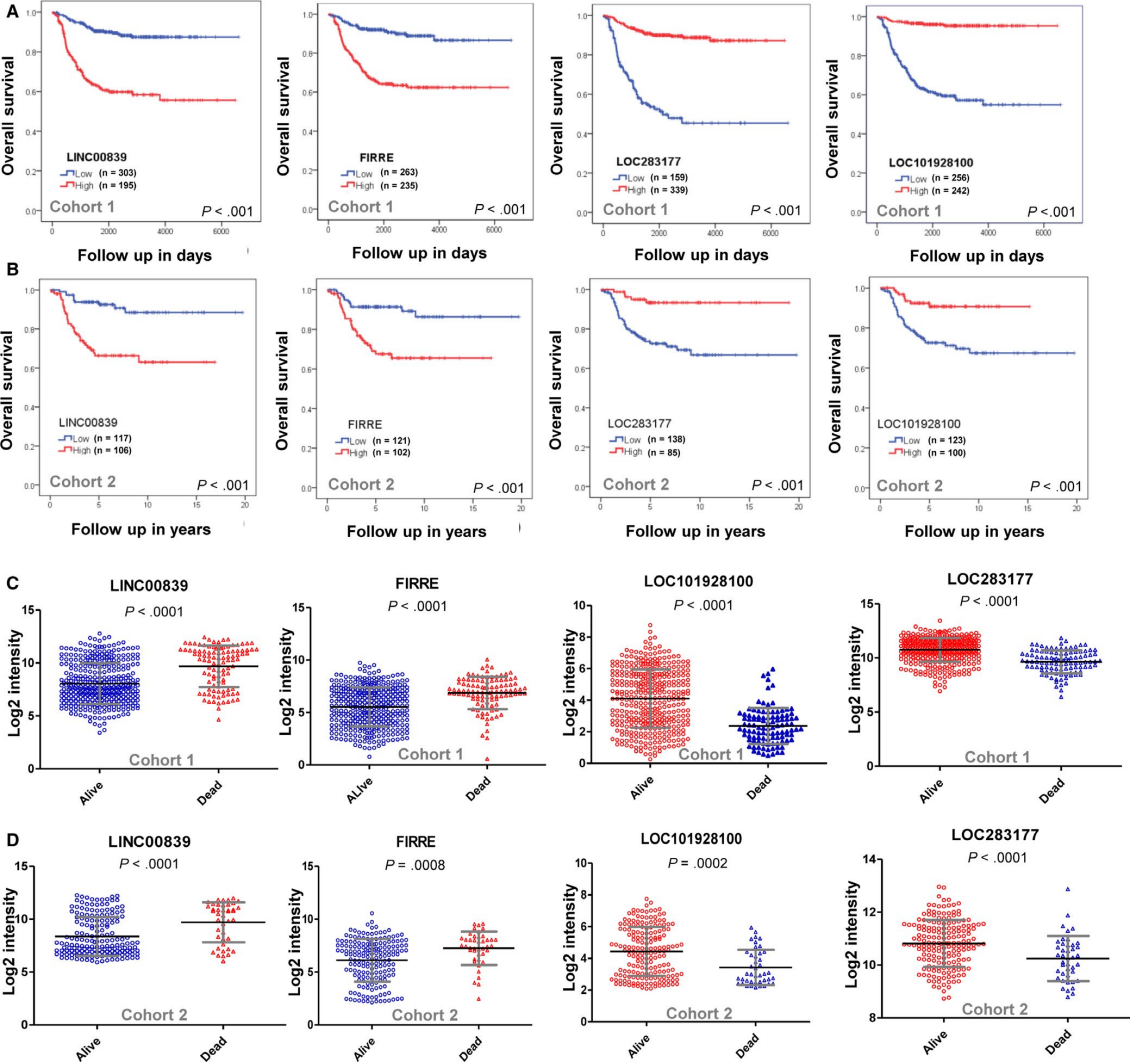
Kaplan‐Meier plots show the OS probability for high expression and low expression of the four lncRNAs in cohort 1 (A) and cohort 2 (B). Scatter plots show the average expression level of the four lncRNAs in the dead patients group and living patients group for cohort 1 (C) and cohort 2 (D). LncRNA, long noncoding RNA; OS, overall survival

Multivariate Cox proportional hazards model survival analysis was performed on cohort 1, and finally four lncRNAs were identified to be significantly (*P* < .001) associated with OS independent with each other (Table S2). These four lncRNAs were also significantly associated with EFS independent with each other (Table S2). Two (FIRRE and LINC00839) of them are “bad survival lncRNAs” and another two (LOC283177 and LOC101928100) are “good survival lncRNAs.” The ROC curves and AUC analyses indicate good performance of each of the four lncRNAs for predicting OS (Figure S1A,B). The expression value which has the highest Youden's index was chosen as the optimal cutoff value for each of the four lncRNAs. According to the optimal cutoff value, the entire cohort was divided into two subgroups (low expression and high expression). Kaplan‐Meier plots revealed that high expression of FIRRE and LINC00839 was significantly (*P* < .001) associated with poor OS, respectively, and high expression of LOC283177 and LOC1019281001 was significantly (*P* < .001) associated with good OS, respectively (Figure 3A).

The identified four lncRNAs were then tested in cohort 2. The ROC curves and AUC analyses also indicate good performance of each of the four lncRNAs for predicting OS in cohort 2 (Figure S1C,D). The expression value which has the highest Youden's index was chosen as the optimal cutoff value and the cohort were divided into two subgroups accordingly. Kaplan‐Meier plots revealed that, in cohort 2, high expression of FIRRE and LINC00839 was also significantly (*P* < .001) associated with poor OS, respectively, and high expression of LOC283177 and LOC1019281001 was also significantly (*P* < .001) associated with good OS, respectively (Figure 3B).

In both of cohort 1 (Figure 3C) and cohort 2 (Figure 3D), the expression level of FIRRE and LINC00839 was significantly higher in NB patients with an outcome of death, while the expression level of LOC010928100 and LOC238711 was significantly lower in NB patients with an outcome of death. The association of the four identified lncRNAs with other clinical characteristics was shown in Figure S2. The expression level of FIRRE and LINC00839 was significantly higher in stage 4 group (Figure S2A), MYCN amplified group (Figure S2B) and age ≥18 months group (Figure S2C); while the expression of LOC010928100 and LOC238711 was significantly higher in stage 4S group (Figure S2A), MYCN nonamplified group (Figure S2B) and age <18 months group (Figure S2C).

### 3.3 Building a four‐lncRNA signature risk score

**FIGURE 4:**
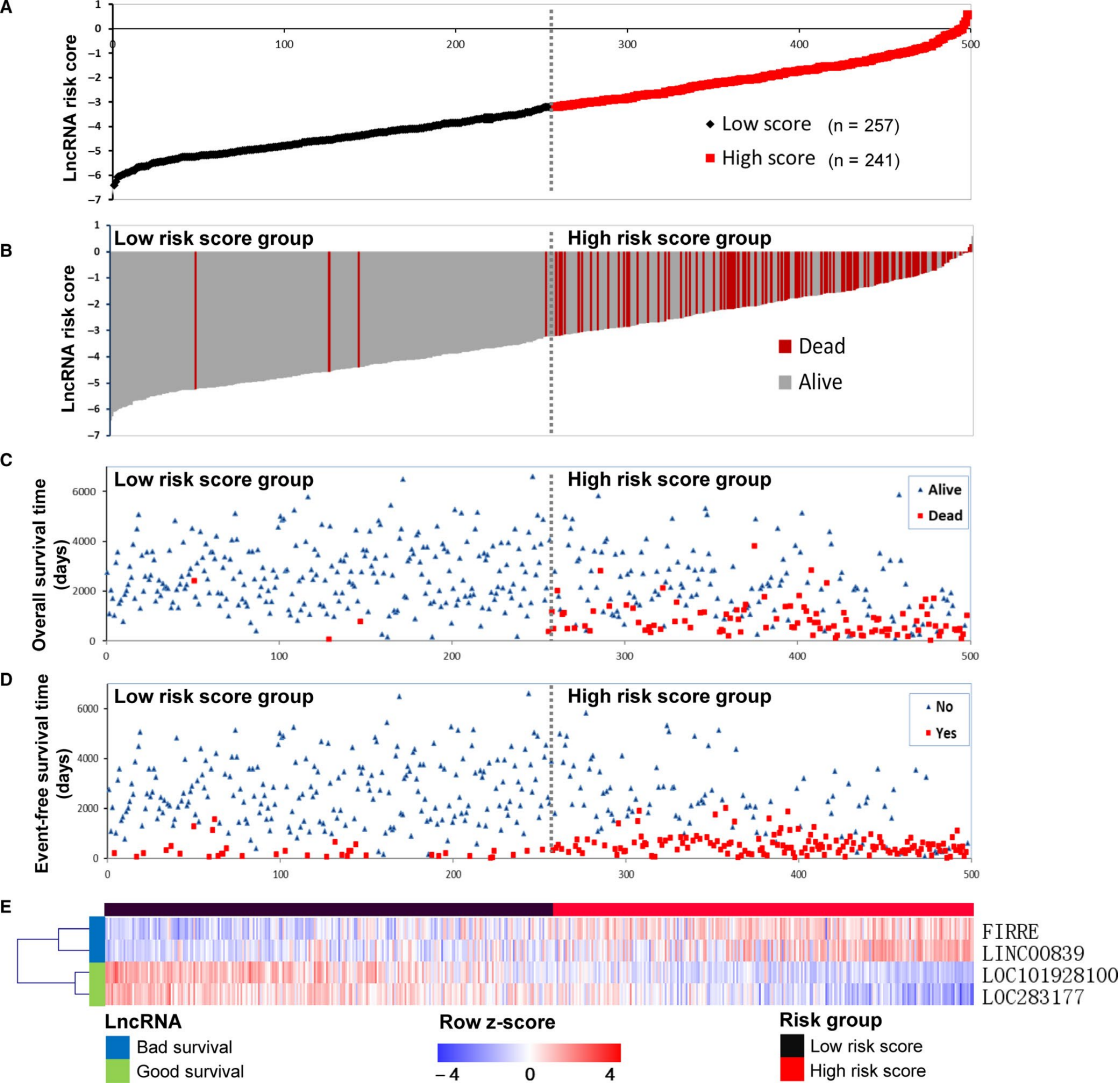
Building a four‐lncRNA signature risk score in the cohort 1. A, Point plot shows high‐ and low‐risk score patients groups divided by the optimal cutoff values and represented by color. Black represents low‐risk score group, and red represents high‐risk score group. B, Waterfall plot of ordered risk scores shows survival status of the patient. Red and gray bars represent patients who died and those who survived, respectively. C, The scatter plot of ordered risk scores shows OS status of each patient. D, The scatter plot of ordered risk scores shows EFS status of each patient. E, Heatmap shows the expression profile of the four‐lncRNA signature. Each column indicates a patient in the low‐risk score group (black) and high‐risk score group (red). Each row represents lncRNAs associated with bad survival (blue) and good survival (green). The lncRNAs were ordered by hierarchical clustering. The expression value of each lncRNA was z‐normalized and is shown with a blue‐red gradient color scale. The gray dashed line in each figure represent the cutoff value point and divided the cohort into two groups with the left part represents low‐risk score group and the right part represents high‐risk score group. EFS, event‐free survival; lncRNA, long noncoding RNA; OS, overall survival

A four‐lncRNA signature risk score was constructed with the multivariate regression coefficients for OS in cohort 1, as follows: risk score = (0.227 × expression value of LINC00839) + (0.276 × expression value of FIRRE) + (−0.522 × expression value of LOC283177) + (−0.341 × expression value of LOC101928100). The four‐lncRNA risk score range from −6.41 to 0.58 (median = −3.3). The ROC curve and AUC analysis indicates good performance for the four‐lncRNA signature risk score (AUC = 0.856; 95% CI: 0.820‐0.892; *P* < .001; Figure S1E). Time‐dependent ROC curve revealed that the prognostic accuracy of the four‐lncRNA in cohort 1 at 3‐, 5‐, and 10‐year were similar (AUC = 0.89, 0.9, and 0.93, respectively; Figure S1G). The risk score which has the highest Youden's index was chosen as the optimal cutoff value (−3.19). The cohort was then separated into low‐risk score group (n = 257) and high‐risk score group (n = 241) according to the optimal cutoff value (Figure 4A). The waterfalls plot (Figure 4B) and scatter plot (Figure 4C) show that more patients in the high‐risk score group died than in the low‐risk score, with only three patients with low‐risk score died during 10 years follow‐up. The scatter plot (Figure 4D) also shows that more patients in the high‐risk score group progressed than in the low‐risk score group. The heatmap (Figure 4E) shows that patients in the high‐risk score group tend to express bad survival lncRNAs and patients in the low‐risk score group tend to express good survival lncRNAs.

The four‐lncRNA signature was tested in cohort 2 for validation using the same risk score formula. A four‐lncRNA signature risk score was constructed for cohort 2. The ROC curve and AUC analysis indicates good performance for the four‐lncRNA signature (AUC = 0.771; 95% CI: 0.701‐0.842; *P* < .001; Figure S1F). Time‐dependent ROC curve revealed that the prognostic accuracy of the four‐lncRNA in cohort 2 at 3‐, 5‐, and 10‐year were similar (AUC = 0.77, 0.78, and 0.77, respectively; Figure S1H).The cohort was then separated into low‐risk score group (n = 152) and high‐risk score group (n = 71) according to the optimal cutoff value (−0.77) (Figure S3A). The waterfalls plot (Figure S3B) and scatter plot (Figure S3C) show that more patients in the high‐risk score group died than in the low‐risk score, with only one patients with low‐risk score died during 10 years follow‐up. The scatter plot (Figure S3D) also shows that more patients in the high‐risk score group progressed than in the low‐risk score group. The heatmap (Figure S3E) shows that patients in the high‐risk score group tend to express bad survival lncRNAs and patients in the low‐risk score group tend to express good survival lncRNAs.

### 3.4 Prognostic role of four‐lncRNA signature risk score

**FIGURE 5:**
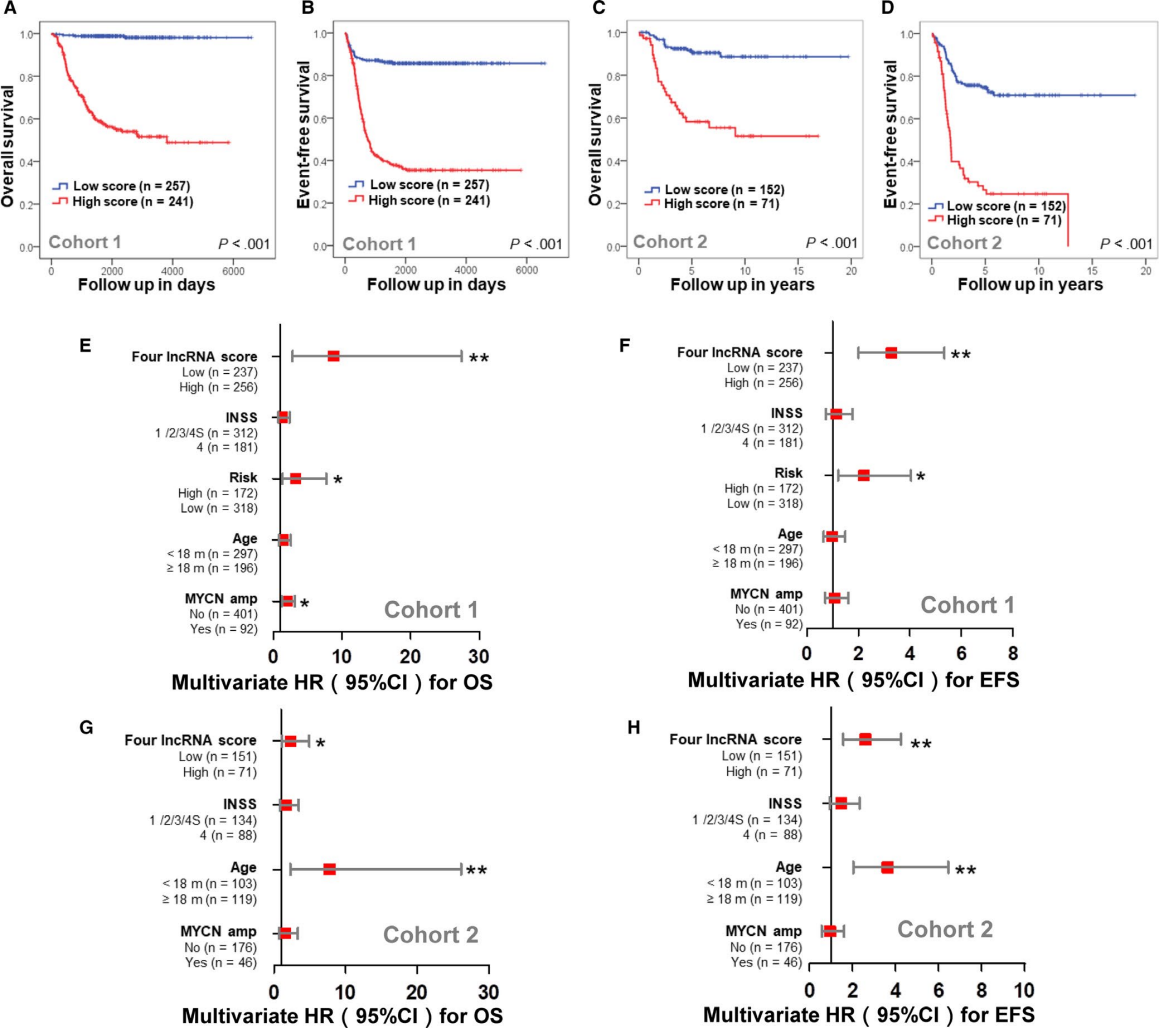
The prognostic role of the four‐lncRNA signature risk score. Kaplan‐Meier plot shows the OS probability (A) and EFS probability (B) for low and high‐risk score groups in cohort 1. Kaplan‐Meier plot shows the OS probability (C) and EFS probability (D) for low and high‐risk score groups in cohort 2. Forest plots show the multivariable Cox analysis of the four‐lncRNA signature for OS (E) and EFS (F) in cohort 1. Forest plots show the multivariable Cox analysis of the four‐lncRNA signature for OS (G) and EFS (H) in cohort 2. All statistical tests were two‐sided. EFS, event‐free survival; lncRNA, long noncoding RNA; OS, overall survival.  *, *P* < .05; **, *P* < .005

Kaplan‐Meier plots show that patients in the high‐risk score group had a significantly poorer OS (Figure 5A) and EFS (Figure 5B) than those in the low‐risk score group. The OS rates at 5 years were 60.6% in the high‐risk score group, compared with 98.8% in the low‐risk score group. The OS rates at 10 years were 58.1% in the high‐risk score group, compared with 98.4% in the low‐risk score group. Only 40.2% of NB patients in the high‐risk score group were event free at 5 years, compared with 86% of patients in the low‐risk score group. Only 39.0% of NB patients in the high‐risk score group were event free at 10 years, compared with 86% of patients in the low‐risk score group. The four‐lncRNA signature also significantly stratified the patients of cohort 2 into two groups for both OS (Figure 5C) and EFS (Figure 5D), respectively.

Multivariate Cox survival analyses including MYCN amplification (nonamplified vs amplified), age status (<18 months vs ≥18 months), risk status (low risk vs high risk), and INSS stage (INSS 1/2/3/4S vs INSS 4) as covariates were performed to evaluate the independent prognostic role of the four‐lncRNA signature (Table S3). In cohort 1, the four‐lncRNA signature (HR = 8.78; 95% CI: 2.82‐27.36, *P* < .001), risk status (HR = 3.24; 95% CI: 1.36‐7.70; *P* = .008), and MYCN status (HR = 1.98; 95% CI: 1.26‐3.13; *P* = .003) were independently associated with OS (Figure 5E); only the four‐lncRNA signature (HR = 3.28; 95% CI: 2.01‐5.35; *P* < .001) and risk status (HR = 2.21; 95% CI: 1.21‐4.05; *P* = .01) were independently associated with EFS (Figure 5F). The cohort 2 did not report the risk status for the samples and the multivariate Cox survival analyses (Table S4) showed that only the four‐lncRNA signature (HR = 2.31; 95% CI: 1.08‐4.92; *P* = .03) and age status (HR = 7.75; 95% CI: 2.03‐26.07; *P* = .001) were independently associated with OS (Figure 5G); the four‐lncRNA signature (HR = 2.59; 95% CI: 1.58‐4.25; *P* < .001) and age status (HR = 3.62; 95% CI: 2.04‐6.44; *P* < .001) were also independently associated with EFS (Figure 5H).

### 3.5 Prognostic role of four‐lncRNA signature risk score within clinical subgroups

**FIGURE 6:**
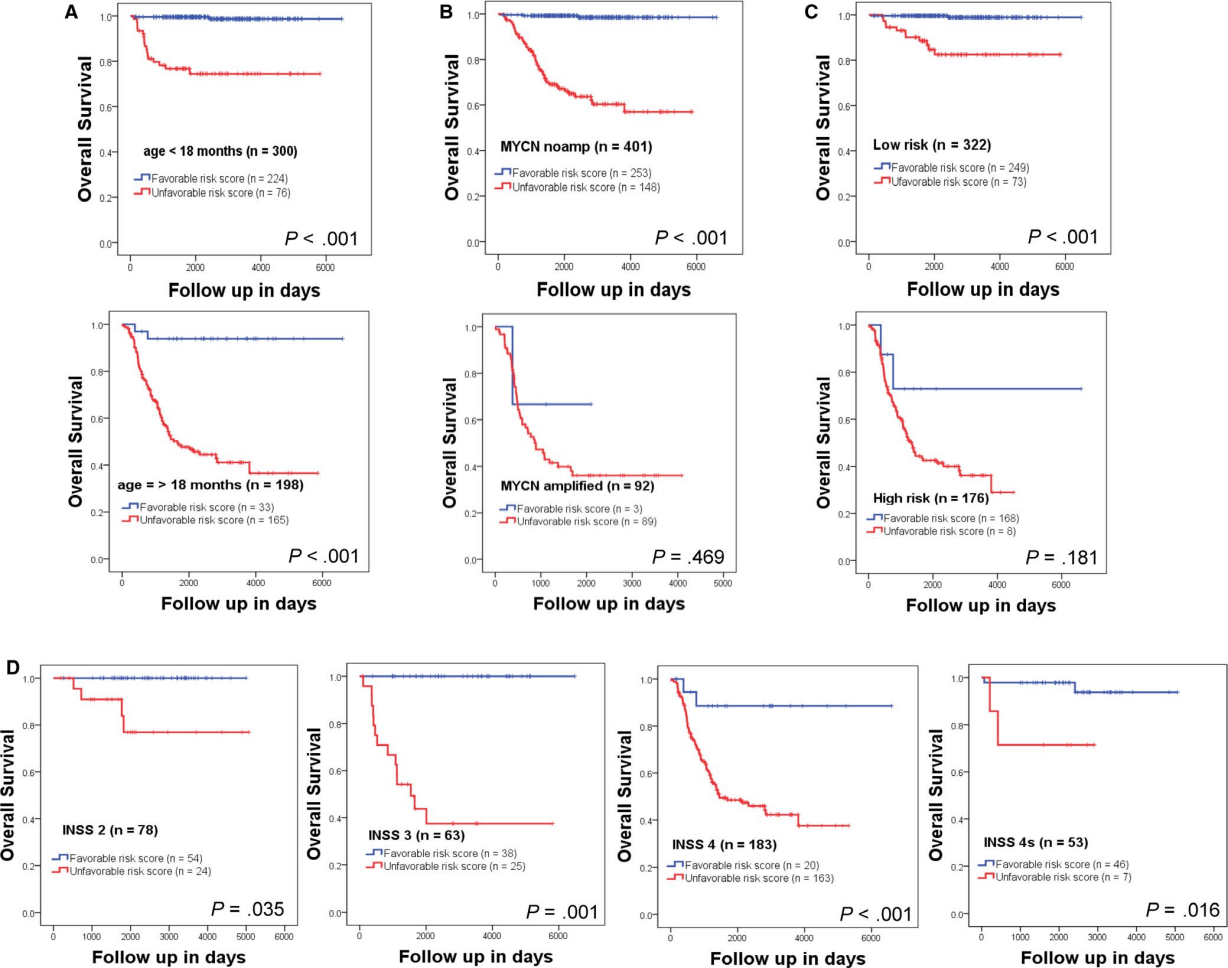
Survival estimates of OS within the clinical risk factors subgroups from the cohort 1. Kaplan‐Meier plots show the OS probability for low and high‐risk score groups in different age subgroups (A), different MYCN amplification status subgroups (B), different risk status subgroups (C), and different INSS stage subgroups (D). The *P* values were obtained using a Mantel log‐rank test (two‐sided). OS, overall survival

In order to corroborate whether the four‐lncRNA signature could predict survival in different clinical subgroups, we performed stratification survival analysis according to MYCN status, age status, risk status, and INSS stage. Within each subgroup, patients were stratified into low‐risk score and high‐risk score subgroup using the optimal cutoff value. In both of the age subgroup (age <18 months and age ≥18 months), patients with high‐risk score had a significantly shorter OS than those with low‐risk score (Figure 6A). In the MYCN nonamplified subgroup, patients with high‐risk score had a significantly shorter OS than those with low‐risk score; however, in the MYCN amplified subgroup, the four‐lncRNA signature failed to significantly stratify patients into two risk score groups for OS (Figure 6B). In the clinically low risk subgroup, patients with high‐risk score had a significantly shorter OS than those with low‐risk score; while in the clinically high‐risk subgroup, the four‐lncRNA risk score failed to significantly stratify patients into two risk groups for OS (Figure 6C). The four‐lncRNA signature also significantly stratified patients into two risk groups for OS within each INSS subgroup (INSS 2, INSS3, INSS 4, and INSS 4S) (Figure 6D). The survival comparison for OS within the INSS 1 subgroup is not shown, as only one patient in this stage died during the follow‐up period. For EFS, the four‐lncRNA signature also successfully significantly stratified the patients into two risk groups within each subgroup except for INSS 2, INSS4S, and MYCN amplified subgroup (Figure S4A‐D).

### 3.6 Correlation of the lncRNAs with MYCN and NTRK1

**FIGURE 7:**
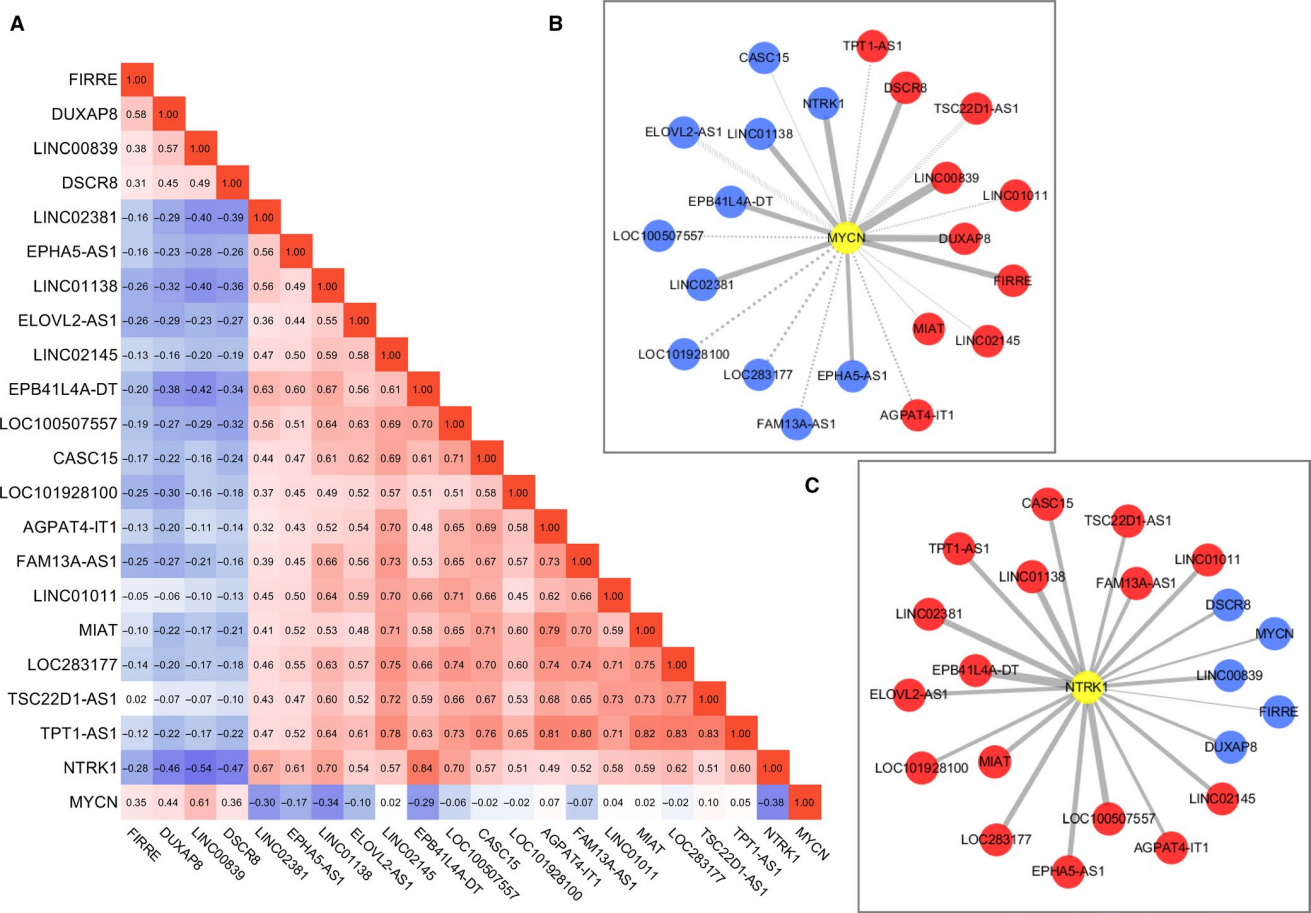
Pearson correlation analysis of the 20 lncRNAs in the cohort 1. A, Pearson correlation analysis matrix shows correlations between each of the 20 lncRNAs. The color scale bar denotes correlation strength, with 1 indicating a positive correlation (red) and −1 indicating a negative correlation (blue). B, The coexpression network of 20 lncRNAs and MYCN. C, The coexpression network of 20 lncRNAs and NTRK1. For the coexpression network, red nodes represent positive correlations with MYCN or NTRK1, and blue nodes represent negative correlations with MYCN or NTRK1. Edge width is proportional to the strength of the correlation. Dashed edges indicate that the correlation is not significant. LncRNA, long noncoding RNA

To understand the regulatory roles of the identified lncRNAs, we performed Pearson correlation analysis between the expression values of the 20 lncRNAs. We found that most of the good survival lncRNAs were positively correlated with each other and negatively correlated with the bad survival lncRNAs (Figure 7A). Most of the bad survival lncRNAs were positively correlated with each other, and negatively correlated with the good survival lncRNAs (Figure 7A). Since MYCN oncogene amplification is one of the most important risk factors of NB, we also investigated the correlation between the expression of those 20 lncRNAs and the expression of MYCN gene. The results showed that the expression of the four bad survival lncRNAs was significantly correlated with the expression of MYCN, respectively (Figure 7B), with LINC00839 has the highest correlation strength (*R* = .61, *P* < .001). However, only four (LINC002381, EPHA5‐AS1, LINC01138, and EPB41L4A‐DT) of the 16 good lncRNAs were negatively correlated with MYCN with statistically significant (*P* < .001) (Figure 7B). Since TrkA (encoded by NTRK1) is a well‐known factor involved in the spontaneous regression of NB, we also investigated the correlation between the expression of those lncRNAs and the expression of NTRK1. It is very interesting to find that all of the 16 good lncRNAs were positively correlated with NTRK1 with statistically significant (*P* < .001), and all of the four bad survival lncRNAs were negatively correlated NTRK1 with statistically significant (*P* < .001) (Figure 7C).

### 3.7 Biological function of the four‐lncRNA signature in NB

The top 1000 correlated genes of each of the lncRNAs were used for the biological function assessment (GO biological process and KEGG pathways). The highest enriched biological function gene sets shared between the two bad survival lncRNAs (FIRRE and LINC00839) are shown in Figure S5A,B, which includes telomere maintenance, DNA replication, DNA repair, RNA localization, RNA degradation, translational elongation, mitochondrion organization, etc The highest enriched biological function gene sets shared between the two good survival lncRNAs (LOC283177 and LOC101928100) are shown in Figure S5C,D, which includes autophagy, establishment of cell polarity, cell projection morphogenesis, neuron migration, synapse organization, axon guidance, MAPK signaling pathway, etc

### 3.8 Genetic alterations of the 20 lncRNAs in NB

The cBioportal platform was utilized to investigate the genetic alterations of those 20 lncRNAs in NB (Figure S6). Four projects with 755 cases reported the mutation data for NB and only one project with 59 NB cases reported the somatic gene copy number data for NB. MYCN gene was found to have somatic gene copy number alteration in 19% of 59 NB cases, and has mutations in 1.2% of 755 NB cases. However, no mutation or somatic gene copy number alteration was discovered for each of the 20 lncRNAs (Figure S6A,B). No mutation or gene copy number alteration of NTRK1 was discovered too. This result indicates that the differential expression of those lncRNAs among different NB samples might not be caused by genetic alterations.

## 4 DISCUSSION

LncRNAs are most commonly defined as RNA transcripts longer than 200 nucleotides with no or little protein‐coding capacity.^31^ LncRNAs are crucial players in a variety of cellular functions, and it is now well recognized that they also play important roles in both oncogenic and tumor‐suppressive pathways in various types of cancers.^17^, ^18^ Increasing evidence also revealed that lncRNAs are implicated in NB. For example, lncRNA CASC15‐S (cancer susceptibility 15) was found to be a tumor suppressor in NB by mediating neural growth and differentiation^20^; lncRNA pancTts‐1 promotes NB progression through hnRNPK‐mediated β‐catenin stabilization^24^; lncRNA lncNB1 promotes tumorigenesis of NB by interacting with ribosomal protein RPL35, and then, enhance E2F1 protein synthesis^32^; our previous studies also showed that lncRNA MYCNOS (MYCN opposite strand) cooperates with transfactor CTCF to promote NB progression through facilitating MYCN gene expression.^32^ Previously, an RNA‐sequence‐based study also identified 20 lncRNAs differentially expressed in both MYCN amplification status (MYCN amplified vs MYCN nonamplified) and risk status (high risk vs low risk).^22^

However, the association between lncRNAs and the spontaneous regression of NB has been rarely investigated. To our knowledge, the present study is the first study aimed at screening for lncRNAs associated with spontaneous regression of NB using microarray data. Since spontaneous regression of NB is most evident in infants with stage 4S disease, we use stage 4S tumors as a surrogate to investigate the mechanisms underlining spontaneous regression like many other investigators have done before.^14^, ^15^, ^16^ We excluded the dead cases in stage 4S to make it better for stage 4S cases to serve as surrogates to NBs underwent spontaneous regression. Actually, only five out 54 patients in stage 4S died in cohort 1 and one out 30 patients died in cohort 2 during 10 years follow‐up. Finally, we identified 20 survival‐related lncRNAs that are differential expressed between those dead cases in stage 4 NB and those living cases in stage 4S NB. The expression level of each of the 20 lncRNAs was significantly associated with not only OS, but also EFS. We also find that the expression of each of the 20 lncRNAs was significantly correlated with the expression of NTRK1. Since TrkA (encoded by NTRK1) is a well‐known factor involved in the spontaneous regression of NB,^25^, ^26^ this result indicates that these lncRNAs might either promote or inhibit the process of NB spontaneous regression.

Among those identified lncRNAs, four (LINC00839, FIRRE, DUXAP8, and DSCR8) are bad survival lncRNAs, and 16 (LINC02381, EPHA5‐AS1, LOC101928100, ELOVL2‐AS1, EPB41L4A‐DT, LINC01138, LINC01011, LOC100507557, CASC15, AGPAT4‐IT1, MIAT, TPT1‐AS1, LOC283177, TSC22D1‐AS1, LINC02145, and FAM13A‐AS1) are good survival lncRNAs. Four lncRNAs (FIRRE, LINC00839, EPB41L4A‐DT, and CASC15) have also previously been identified to be prognostic of survival in NB patients by the study conducted by Sahu et al,^22^ which focus on identifying lncRNAs differentially expressed in different MYCN amplification group and different risk group. CASC15‐S has already been found to be a tumor suppressor in NB as mentioned above,^20^ which is consistent with our findings. MIAT (Myocardial Infarction Associated Transcript) has recently been found to possess tumor‐promoting properties in glioblastoma and NB, which is contrary to our findings and need further investigation.^33^ The rest of the other lncRNAs identified in this study have not been reported to be associated with NB before. Five (LICN00839, DUXAP8, DSCR8, LINC01138, LOC283177, and TPT1‐AS1) of them have been reported to be involved in other malignancies.^34^, ^35^, ^36^, ^37^, ^38^, ^39^, ^40^, ^41^, ^42^, ^43^ For example, DUXAP8 function as an oncogene and promote cell proliferation and invasion in multiple types of malignancies including non‐small cell lung cancer, renal cell carcinoma, colorectal cancer, and oesophageal squamous cell cancer^34^, ^35^, ^36^, ^37^; DSCR 8 acts as a molecular sponge for miR‐485‐5p and activate Wnt/β‐catenin signal pathway in hepatocellular carcinoma^38^; TPT1‐AS1 promotes tumorigenesis and metastasis in cervical cancer and epithelial ovarian cancer.^42^, ^43^ However, the exact role of these lncRNAs in NB and their underlining mechanisms need to be further investigated.

Previous study revealed that stage 4 NBs were 90% near‐diploid/tetraploid, 77% had 1p LOH (50% 1p36), 23% 11q, and/or 14q LOH, and 47% had 7q gain; while stage 4S were 90% near‐triploid and LOH was restricted to 11q.^10^ A significant portion of differentially expressed genes mapped to chromosome 1 (90% with higher expression in stage 4S), and chromosome 11 (91% with higher expression in stage 4).^10^ The results of our present study are somewhat different. Of the 16 lncRNAs which are highly expressed in stage 4S, five (31.25%) map to chromosome 6, two (12.5%) map to chromosome 4, two (12.5%) mapped to chromosome 5, one (6.25%) map to 11q25, one (6.25%) map to 1q21, and one (6.25%) map to 12p13. Of the four lncRNAs which are highly expressed in stage 4, one (25%) map to 10q11, one (25%) map to 21q22, one (25%) map to 22q11, and one (25%) map to Xq26. Investigation of 755 NB cases with mutation data and 59 NB cases with somatic gene copy number data revealed no mutation or somatic gene copy number alteration for each of the 20 lncRNAs. This result suggests that the differential expression of these 20 lncRNA might not be caused by genetic alterations. However, this result should be interpreted with caution since the provided genetic alteration data are limited. More studies are warranted to figure out whether these differentially expressed lncRNAs have genetic alterations or not. In addition, genetic alterations outside those lncRNA genes (in cis‐acting or transacting models) might be possible causes accounting for their altered expression. Further relevant researches are needed to clarify the mechanisms of their altered expression.

The identified 20 lncRNAs successfully divided each of the two independent cohorts into two different clusters, with one cluster has good survival outcome and one cluster has bad survival outcome. In order to simplify the lncRNA signature, the multivariable survival analysis was performed, and only four lncRNAs which are significantly associated with survival independently are included in the lncRNA signature risk score. The four‐lncRNA signature performed as well as the total 20 lncRNAs in predicting survival. As for the four‐lncRNA signature, the OS rates at 10 years were 58.1% in the high‐risk score group compared with 98.4% in the low‐risk score group; while for the 20 lncRNAs, the OS rates at 10 years were 59.4% in the bad survival cluster compared with 98.8% in good survival cluster. The four‐lncRNA signature also successfully stratified patients into two risk groups for OS prediction within each INSS subgroup (INSS 2, INSS3, INSS 4, and INSS 4S) with the low‐risk score groups have a good survival and the high‐risk score group have a bad survival. It has to be mentioned that the four‐lncRNA signature failed to stratify patients into two risk group for MYCN amplified subgroup and high‐risk subgroup. These results indicated that the four‐lncRNA signature might not be helpful for those patients with these high‐risk factors. However, the four‐lncRNA signature successfully stratified patients into two risk group for MYCN nonamplified subgroup and low risk subgroup, which meant that the four‐lncRNA signature is helpful in screening out those high‐risk patients which previously considered being at low risk for more aggressive therapy. The prognostic role of the four‐lncRNA signature is also independent with other well‐established clinical risk factors such as MYCN amplification (nonamplified vs amplified), age status (<18 months vs ≥18 months), risk status (low risk vs high risk), and INSS stage (INSS 1/2/3/4S vs INSS 4). These results strongly suggest the use of this four‐lncRNA signature as clinical biomarker for risk stratification and therapeutic guidance.

Gene function (GO biological process and KEGG pathways) annotation revealed that the two bad survival lncRNAs (FIRRE and LINC00839) of the four‐lncRNA signature are involved in the processes, which promote cancer progression such as telomere maintenance, DNA replication, and DNA repair. It seems that metabolism processes also play an important role in NB since the function prediction identified many metabolism processes such as purine metabolism, tRNA metabolic process, nucleotide metabolic process, cysteine, methionine metabolism, etc In fact, purines are basic components of nucleotides in cell proliferation, thus, impaired purine metabolism is associated with the progression of cancer.^44^ However, how does purine metabolism affect NB progression or regression is unknown. The two good survival lncRNAs (LOC283177 and LOC101928100) are involved in the processes of neural differentiation and maturation, such as cell projection morphogenesis, neuron migration, synapse organization, dorso‐ventral axis formation, axon guidance, etc This result indicates that the two good lncRNAs might facilitate the spontaneous regression of NB by promoting neural differentiation and maturation. Undoubtedly, further investigations are needed to clarify how these lncRNAs affect the process of spontaneous regression in NB.

There are also some limitations in our study as follows:

First, only two independent cohorts were included in our study. Initially, we tried to include the GEO datasets GSE49711 in our study. The cohort in GSE49711 is the same with that in GSE49710, while GSE49711 generated gene expression profiles using RNA deep‐sequencing. However, GSE49711 only provided processed data which contains only a small number of lncRNAs. After differentially expression analysis, no lncRNAs identified from GSE49711 were shared with GSE49710. We also tried to include datasets generated from other microarray platforms, for example, the GSE16476 (n = 88) from Affymetrix platform (Affymetrix Human Genome U133 Plus 2.0 Array). However, we also found that only few numbers of identified lncRNAs were shared between those cohorts. We think that the small sample size of cohort GSE16476 (only 12 cases of stage 4S NB) and different range of lncRNAs in the two microarray platforms might be the possible causes. Thus, only the two Agilent microarray datasets were included in our study in order to keep the data consistency.
Second, there are also some differences between cohort 1 and cohort 2. For example, the sample size of cohort 2 (n = 223) is relatively smaller than cohort 1 (n = 498), the age distribution of patients in cohort 2 is significantly different from that in cohort 1, and the distribution of tumor stage in cohort 2 is also significantly different from that in cohort 1 (Table 1). Considering these differences, we constructed a risk score and determined the optimal cutoff value for cohort 2 separately. Since the analysis of Youden's index revealed different cutoff values for the two cohorts, we think it might also be reasonable for them to use their own optimal cutoff value. The difference between cohort 1 and cohort 2 might also responsible for the difference of multivariate survival analysis results between these two cohorts. Another limitation is that the risk status of the samples is not available for cohort 2, which might also contribute to the discrepancy of multivariate analysis. Despite these limitations, the results of cohort 2 also corroborated the results of cohort 1. The results still revealed that the four‐lncRNA signature is independently associated with both OS and EFS in both of the two cohorts. Of course, more researches with large sample size are needed to verify this four‐lncRNA signature.
Third, this four‐lncRNA signature was constructed according to OS and aimed to predicting OS of NB patients. As is shown in Figure 4C, the four‐lncRNA signature has a good performance in predicting OS in cohort 1 with only few patients died in the low‐risk score group. However, in predicting EFS, the performance of the four‐lncRNA signature is not as well as in predicting OS (Figure 4D). The predictive role of the four‐lncRNA signature for EFS is also not as well as for OS in cohort 2 (Figure 3C,D). Thus, it would be better to generate a unique lncRNA signature according to EFS to predicting EFS of NB patients.
Fourth, spontaneous regression of NB is not restricted to stage 4S and not all cases in stage 4S underwent spontaneous regression. However, many other investigators have used stage 4S tumors as a surrogate to investigate the mechanisms responsible for spontaneous regression. We also excluded the dead cases in stage 4S to make it better for stage 4S cases to serve as surrogates. We hope that our research has some enlightening significance for the study of spontaneous regression of NB.
Finally, we did not perform experimental studies to corroborate our findings. The specific function of the identified lncRNAs as well as their underlining mechanisms in NB progression or regression needs to be investigated by further experimental researches. Despite these drawbacks, two independent cohorts with large sample sizes were used in this study to corroborate the results, which provide a high level of confidence.

In conclusion, we identified 20 survival‐related lncRNAs differentially expressed between the dead cases samples in stage 4 NB and the living cases samples in stage 4S NB. The expression of each of these 20 lncRNAs was significantly correlated with the expression of NTRK1, indicating the involvement of those lncRNAs with the spontaneous regression of NB. We also built a four‐lncRNA signature risk score which has a good performance in predicting survival independent with MYCN amplification, age status, risk status, and INSS stage. The four‐lncRNA signature also successfully stratified patients into two risk groups within each INSS subgroup (INSS 2, INSS3, INSS 4, and INSS 4S). These results strongly suggest the use of this four‐lncRNA signature as clinical biomarker for risk stratification or therapeutic guidance in NB.

## CONFLICT OF INTEREST

The authors declare that the research was conducted in the absence of any commercial or financial relationships that could be construed as a potential conflict of interest.

## AUTHOR CONTRIBUTIONS

XZ conceived and designed the study. XYM, EHF, and XZ performed the data analysis. XYM and EHF wrote the original manuscript. XZ and JXF supervised and revised the manuscript. XZ and JXF provided funding acquisition. XYM and EHF contributed equally to this work. All authors read and approved the final manuscript.

## Supporting information

Fig S1

Fig S2

Fig S3

Fig S4

Fig S5

Fig S6

Tables S1‐S4

## Data Availability

The datasets GEO49710 used in this study is public available data and can be found in the GEO datasets (https://www.ncbi.nlm.nih.gov/geo/query/acc.cgi?acc=GSE49710). The datasets E‐MTAB‐8248 used in this study is public available data and can be found in ArrayExpress (https://www.ebi.ac.uk/arrayexpress/experiments/E‐MTAB‐8248/). All other data generated or analyzed during this study are included in the manuscript and it is Supporting Information.
