# Supplementary figures and images for: Identification of prognostic long noncoding RNAs associated with spontaneous regression of neuroblastoma
Source: Cancer Med. 2020 Mar 26;9(11):3800–15. doi: 10.1002/cam4.3022 (PMC7286466; doi:10.1002/cam4.3022)

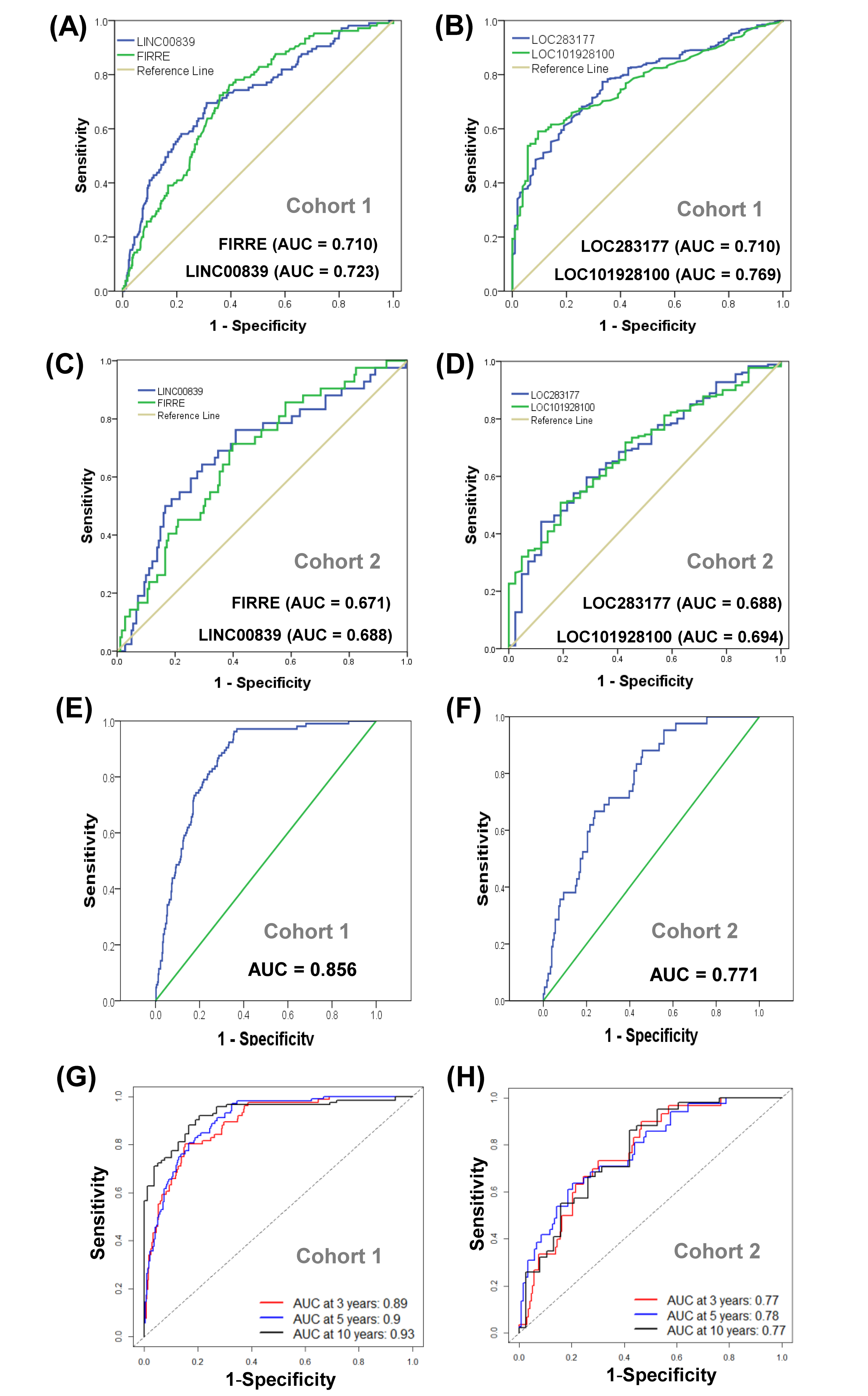

Supplement: Supplementary file 1 — Fig S1 [file CAM4-9-3800-s001.tif]

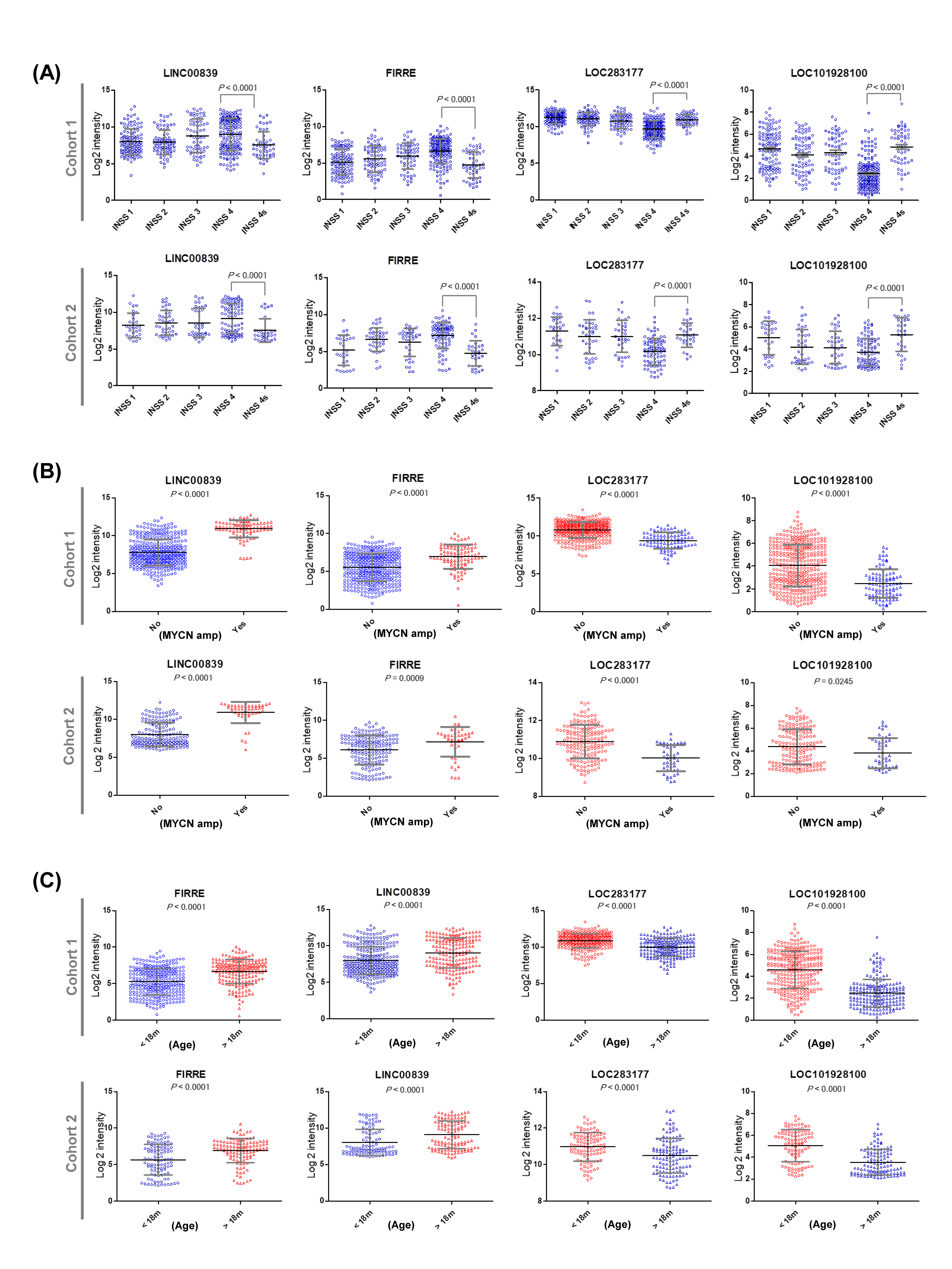

Supplement: Supplementary file 2 — Fig S2 [file CAM4-9-3800-s002.tif]

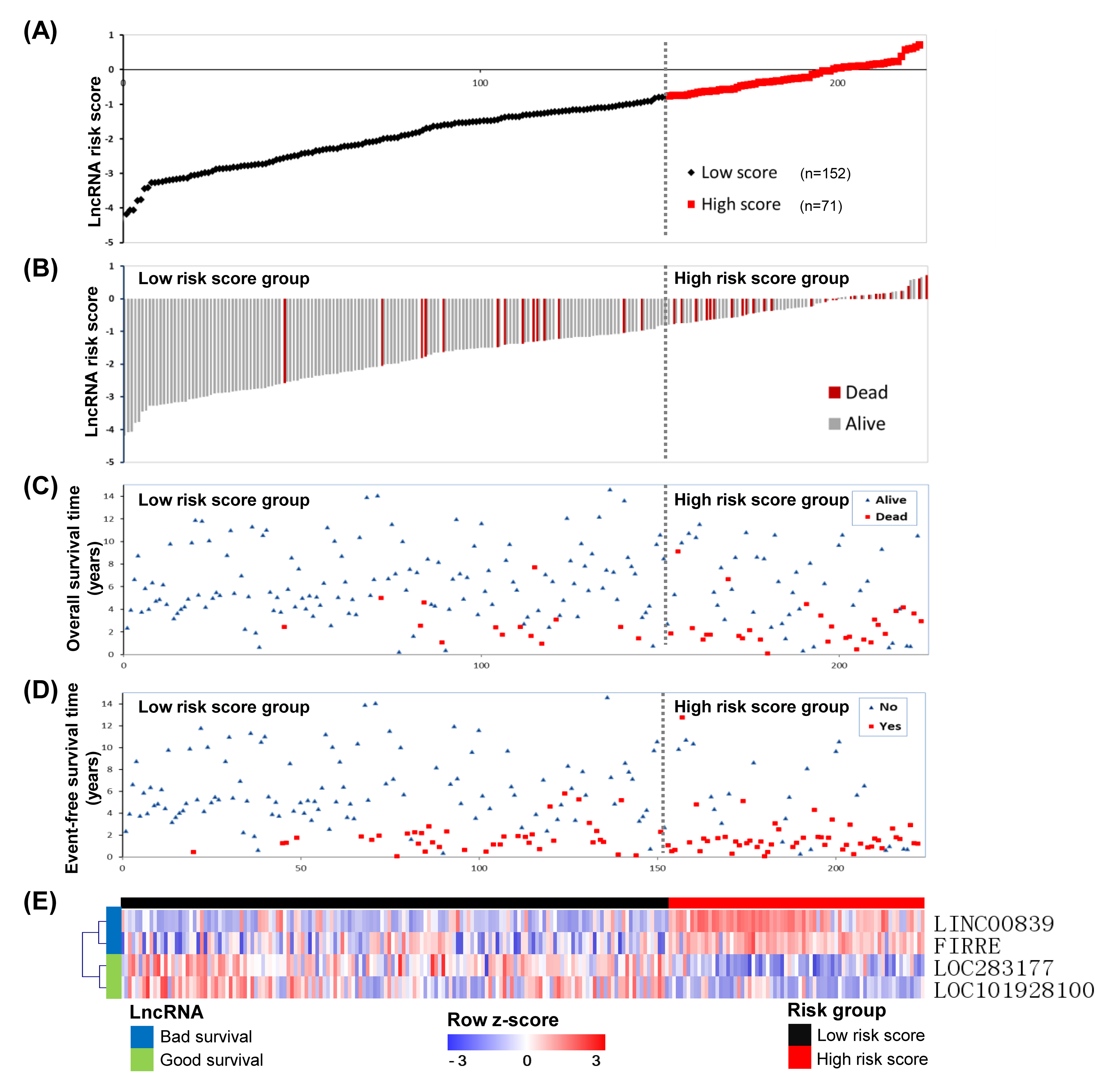

Supplement: Supplementary file 3 — Fig S3 [file CAM4-9-3800-s003.tif]

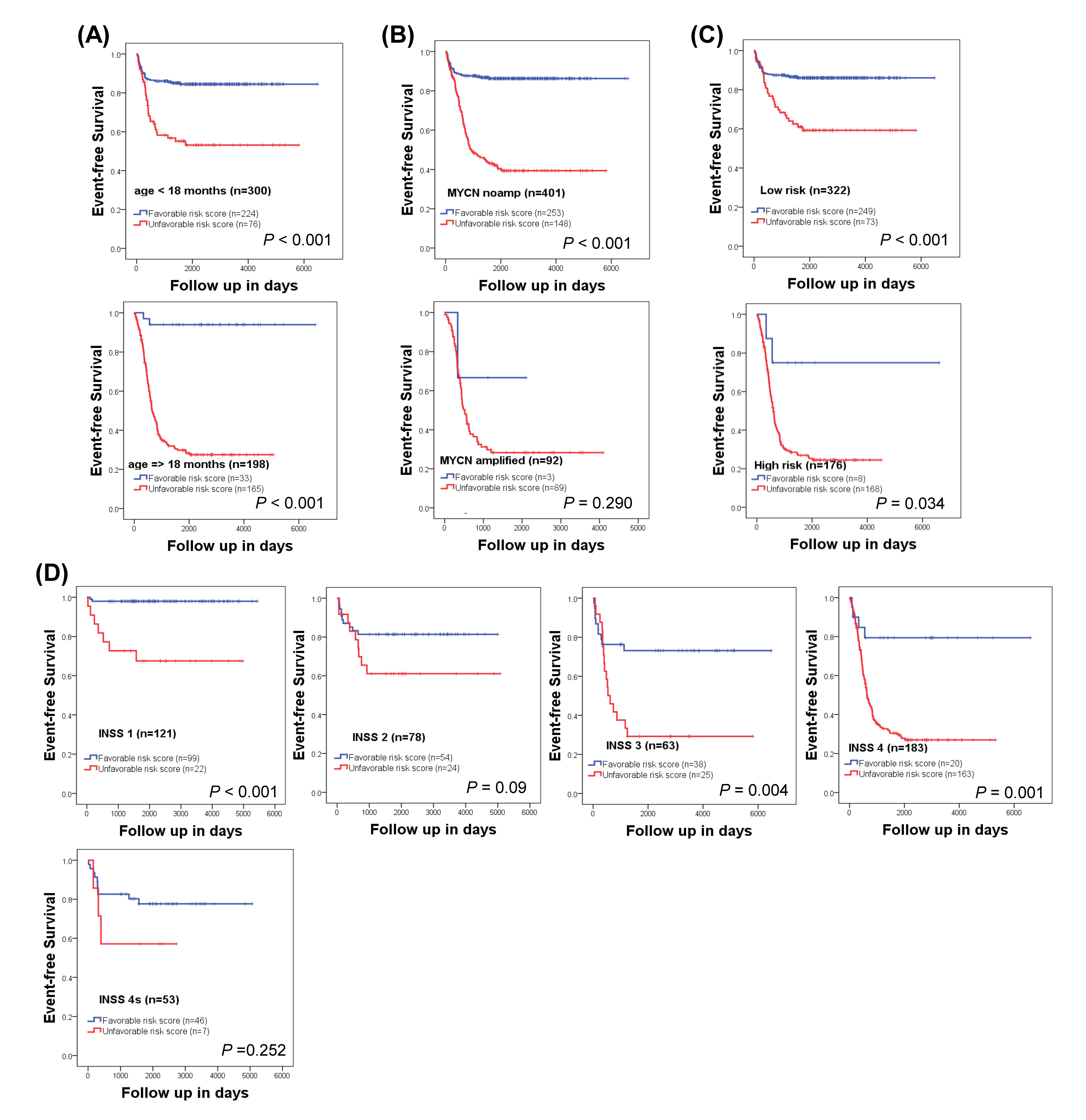

Supplement: Supplementary file 4 — Fig S4 [file CAM4-9-3800-s004.tif]

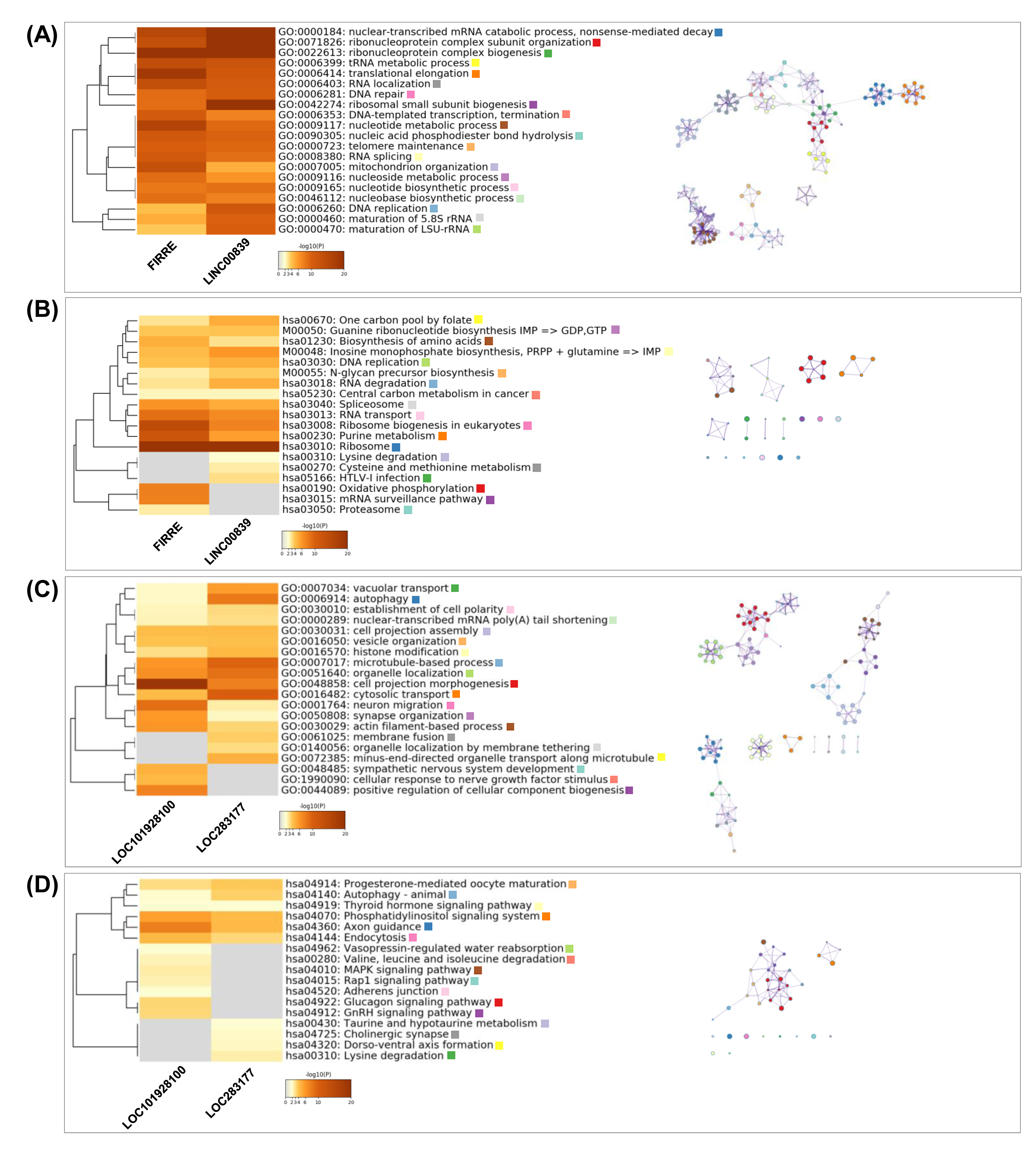

Supplement: Supplementary file 5 — Fig S5 [file CAM4-9-3800-s005.tif]

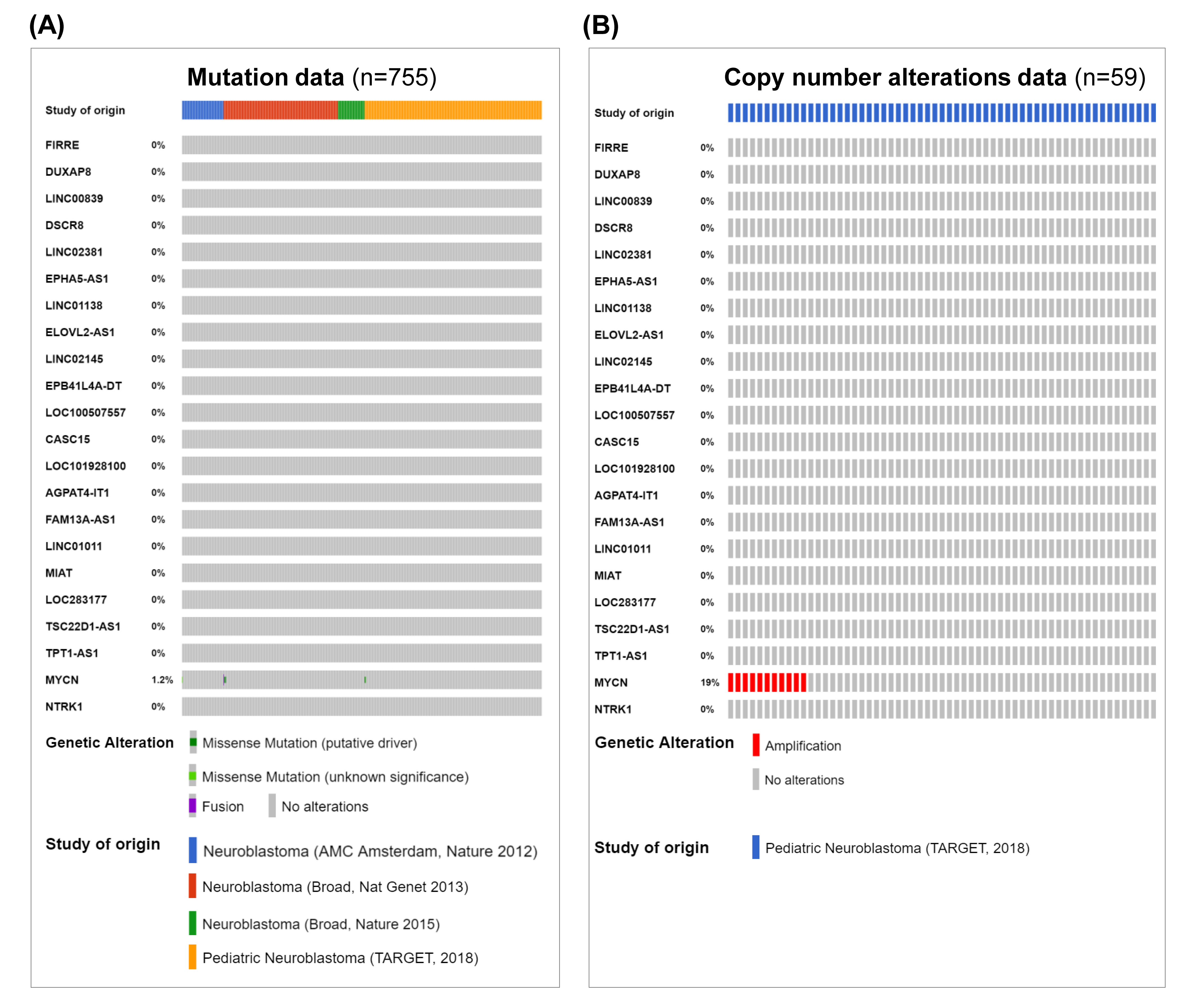

Supplement: Supplementary file 6 — Fig S6 [file CAM4-9-3800-s006.tif]
