# Supplementary material for: Identification of prognostic long noncoding RNAs associated with spontaneous regression of neuroblastoma
Source: Cancer Med. 2020 Mar 26;9(11):3800–15. doi: 10.1002/cam4.3022 (PMC7286466; doi:10.1002/cam4.3022)
Supplement: Supplementary file 7 — Tables S1‐S4 [file CAM4-9-3800-s007.docx]

Supporting information

## Supporting Figures


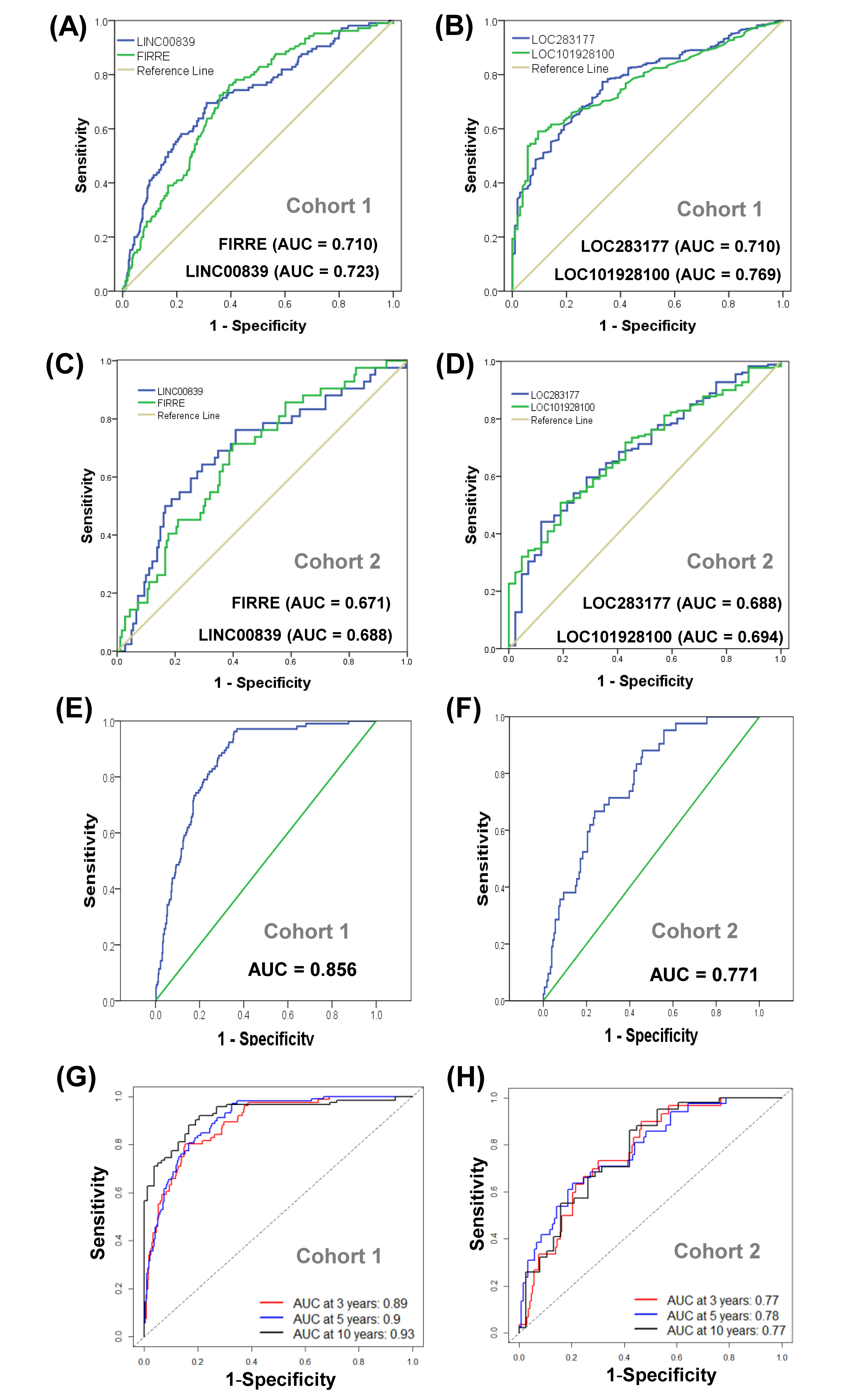


**Figure S1.** Receiver operating characteristic (ROC) curve analysis of overall survival (OS) prediction by the four-lncRNA signature. **(A)** ROC analysis of OS prediction by the two bad survival lncRNAs in cohort 1. **(B)** ROC analysis of OS prediction by the two good survival lncRNAs in cohort 1. **(C)** ROC analysis of OS prediction by the two bad survival lncRNAs in cohort 2. **(D)** ROC analysis of OS prediction by the two good survival lncRNAs in cohort 2. **(E)** ROC analysis of OS prediction by the four-lncRNA signature in cohort 1. **(F)** ROC analysis of OS prediction by the four-lncRNA signature in cohort 2. **(G)** Time-dependent ROC analysis of OS prediction by the four-lncRNA signature in cohort 1. **(H)** Time-dependent ROC analysis of OS prediction by the four-lncRNA signature in cohort 2. lncRNA = long non-coding RNA; ROC = receiver operating characteristic; OS = overall survival.


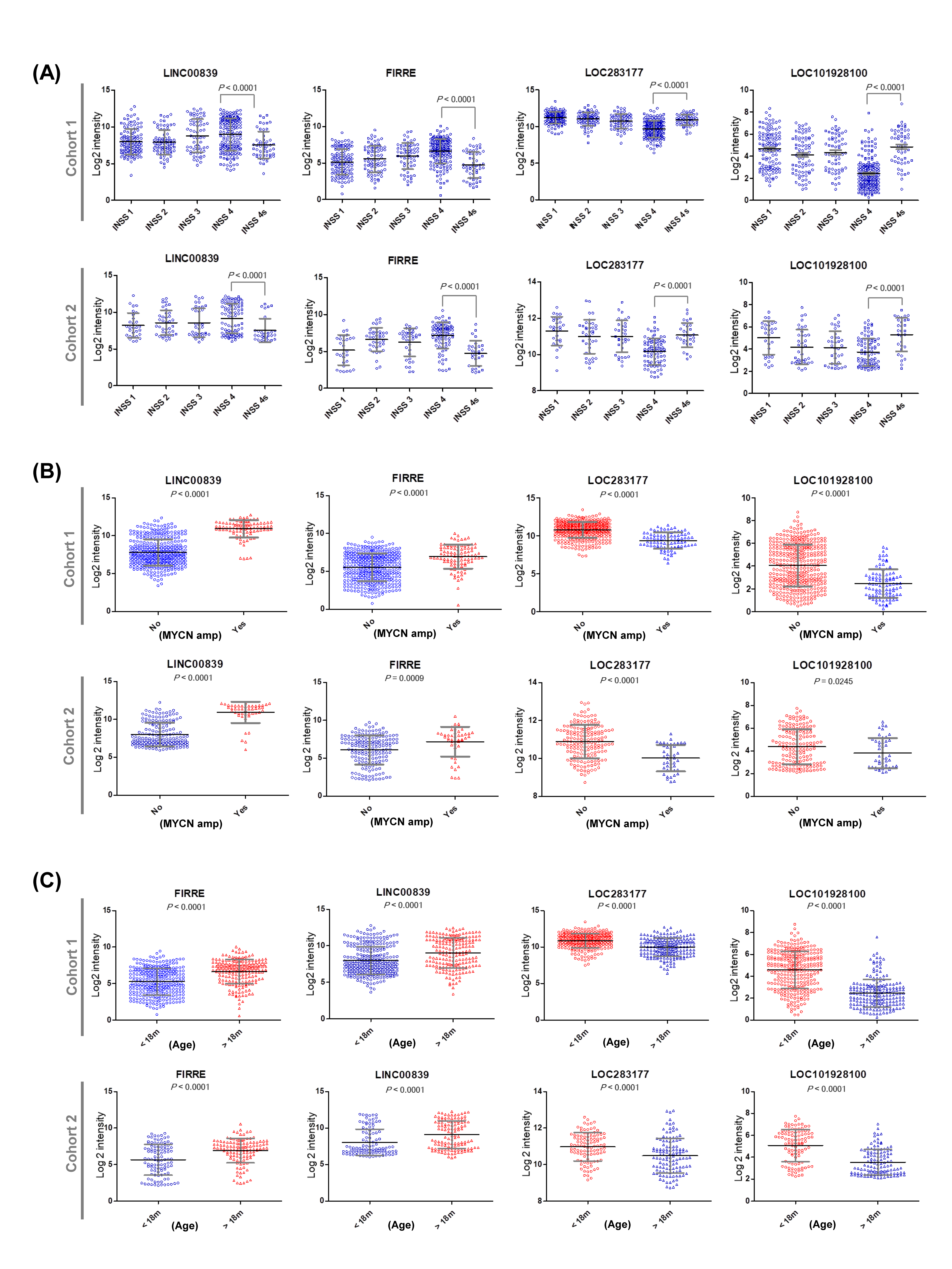


**Figure S2.** (A) Scatter plots show the average expression level of the four lncRNAs in each INSS stage group. (B) Scatter plots show the average expression level of the four lncRNAs in the MYCN amplified group and MYCN non-amplified group. (C) Scatter plots show the average expression level of the four lncRNAs in the age < 18 months group and age ≥ 18 months group. LncRNA = long non-coding RNA.





**Figure S3.** Building a four-lncRNA signature risk score in cohort 2. **(A)** Point plot shows high and low risk score patients groups divided by the optimal cut-off value and represented by color. **Black** represents low risk score group, and **red** represents high risk score group. **(B)** Waterfall plot of ordered risk scores shows survival status of the patient. **Red and gray bars** represent patients who died and those who survived, respectively. **(C)** The scatter plot of ordered risk scores shows overall survival (OS) status of each patient. **(D)** The scatter plot of ordered risk scores shows event-free survival (EFS) status of each patient. **(E)** Heat map shows the expression profile of the four- lncRNA signature. **Each column** indicates a patient in the low risk score group (**black**) and high risk score group (red). **Each row** represents lncRNAs associated with bad survival (**blue**) and good survival (**green**). The lncRNAs were ordered by hierarchical clustering. The expression value of each lncRNA was z-normalized and is shown with a blue-red gradient **color scale**. The gray dashed line in each figure represent the cut-off value point and divided the cohort into two groups with the left part represents low risk score group and the right part represents high risk score group. LncRNA = long non-coding RNA; OS= overall survival; EFS = event-free survival.


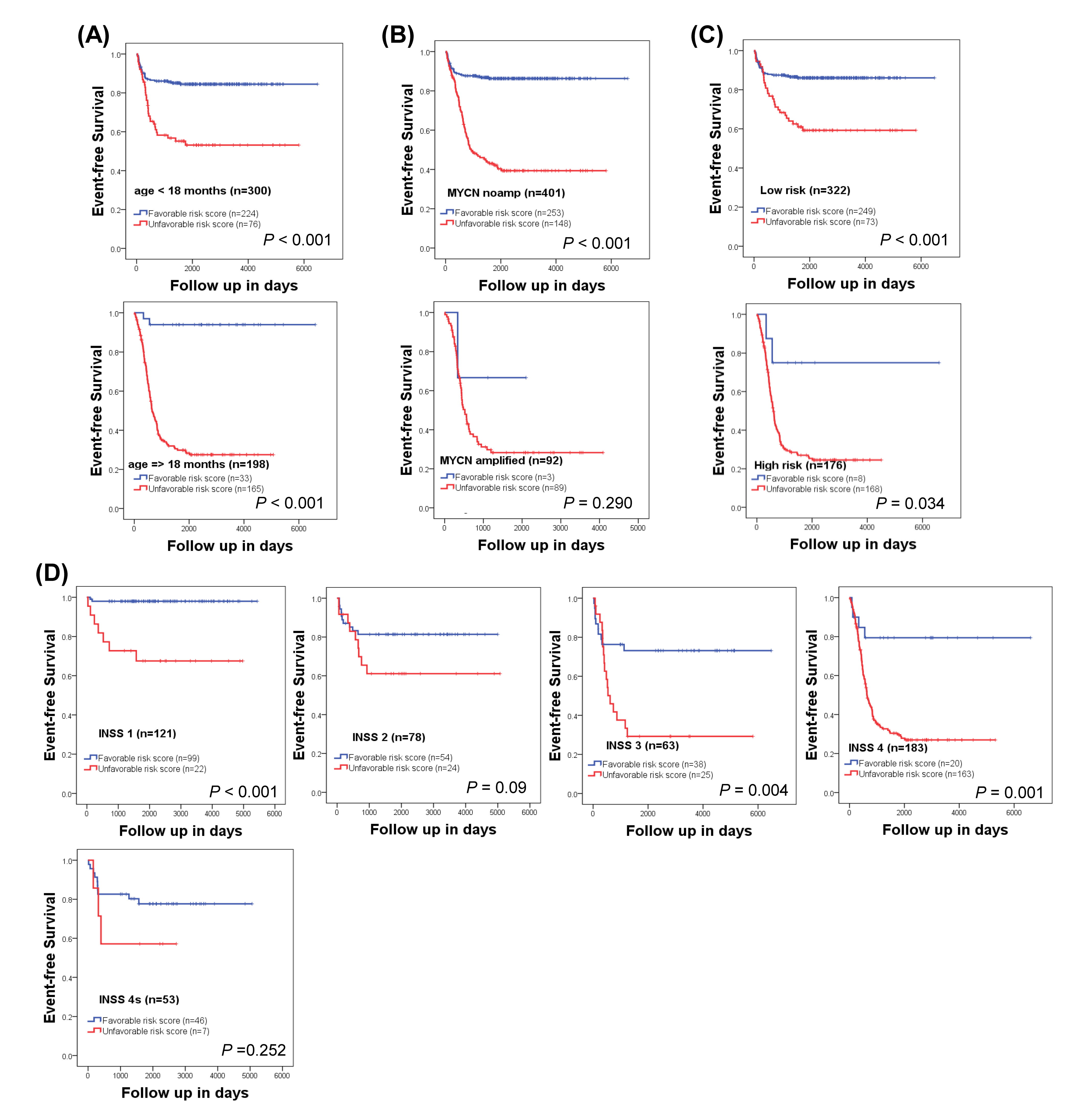


**Figure S4.** Survival estimates of event-free survival (EFS) within the clinical risk factors subgroups from cohort 1. **(A)** Kaplan-Meier plots show the EFS probability for low and high risk score groups in different age subgroups. **(B)** Kaplan-Meier plots show the EFS probability for low and high risk score groups in different MYCN amplification status subgroups. **(C)** Kaplan-Meier plots show the EFS probability for low and high risk score groups in different risk status subgroups. **(D)** Kaplan-Meier plots show the EFS probability for low and high risk score groups in different INSS stage subgroups. The p-values were obtained using a Mantel log-rank test (two-sided). EFS = event-free survival.


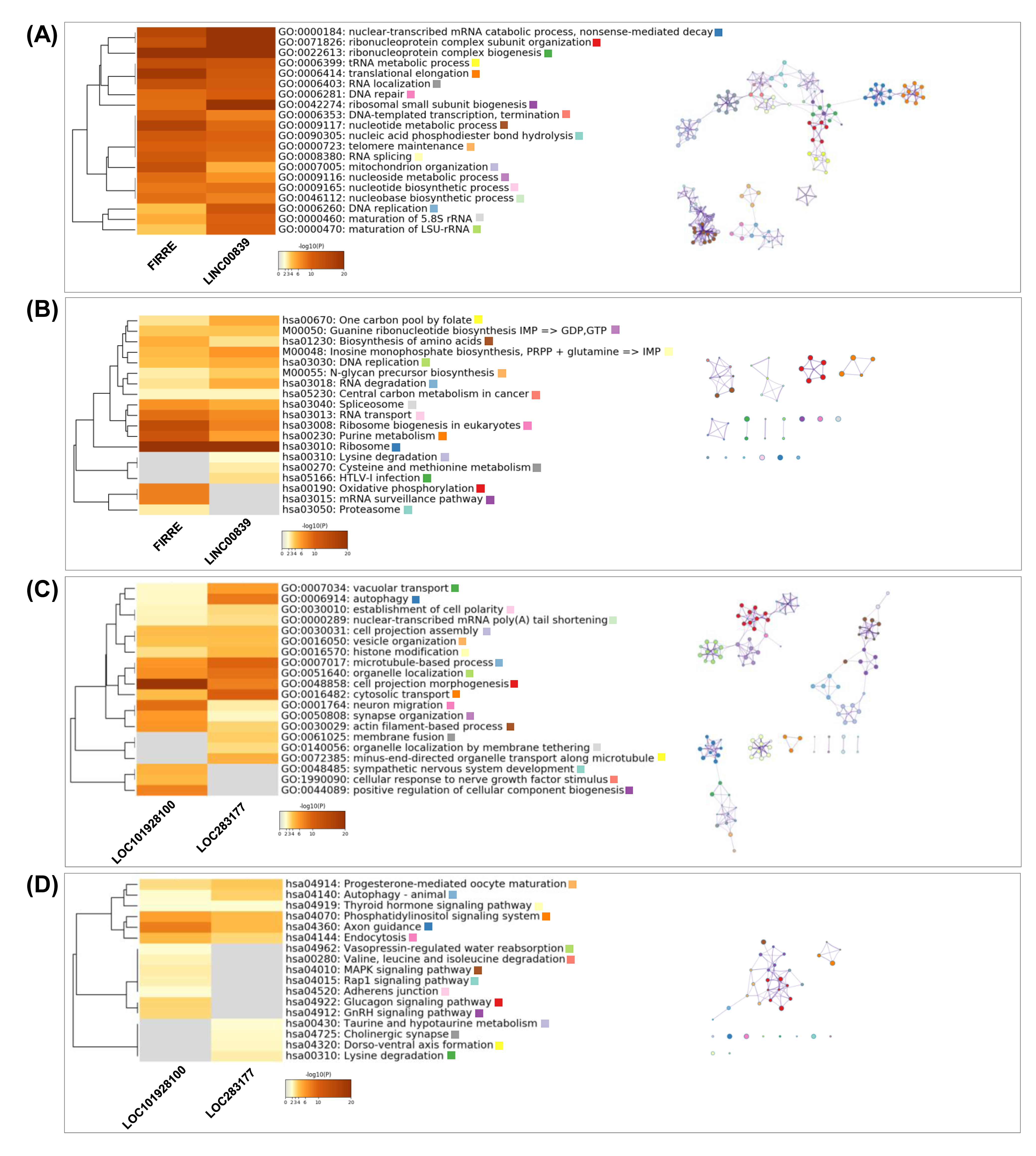


**Figure S5.** Functional prediction analysis of the four-lncRNA signature in neuroblastoma patients. Heatmap and network show the top enriched Gene Ontology (GO) biological process (A) and the top enriched KEGG-pathways (B) shared between the two bad survival lncRNAs (FIRRE and LINC00839). Heatmap and network show the top enriched Gene Ontology (GO) biological process (C) and the top enriched KEGG-pathways (D) shared between the two good survival lncRNAs (LOC283177 and LOC101928100). For the gene sets network, node size is proportional to the normalized enrichment score, and node color represents different gene sets and is in accordance with the gene sets terms labeled with the same color. Biologically related gene sets tend to form clusters by edges. LncRNA = long non-coding RNA


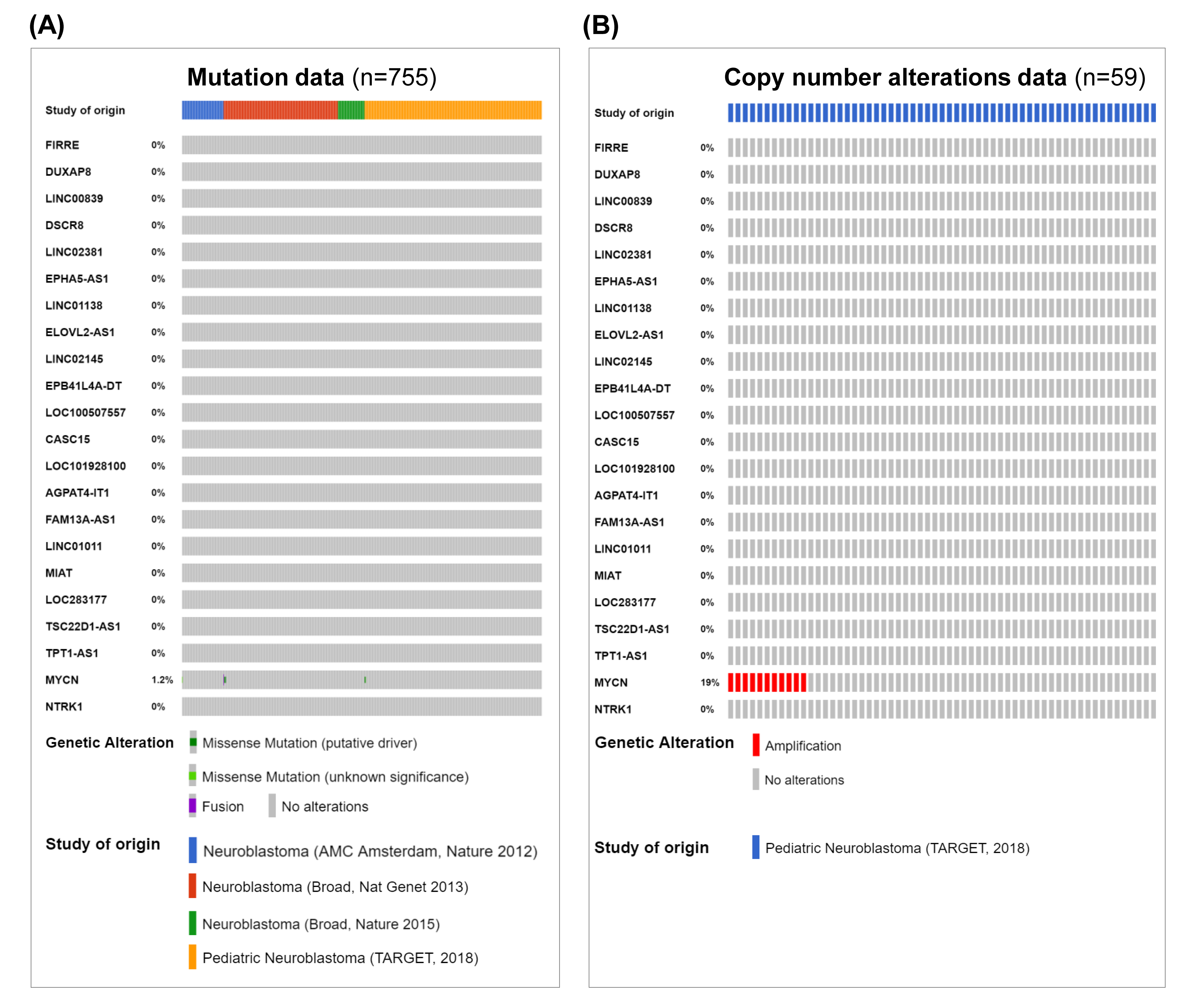


**Figure S6.** Genetic alterations of the lncRNAs in neuroblastoma. (A) Gene mutation data of the lncRNAs from four projects with 755 NB cases. (B) Gene copy number alteration data of the lncRNAs from one project with 59 NB cases. LncRNA = long non-coding RNA.

## Supporting Table

**Table S1.** Univariate Cox proportional hazards model survival analysis of the 20 identified long non-coding RNAs.

| **LncRNA** | **Overall survival for cohort 1** | | | |  | **Event-free survival for cohort 1** | | | |  | **Overall survival for cohort 2** | | | |  | **Event-free survival for cohort 2** | | | |
| --- | --- | --- | --- | --- | --- | --- | --- | --- | --- | --- | --- | --- | --- | --- | --- | --- | --- | --- | --- |
|  | **HR** | **95%CI** | **Z** | ***P* value** |  | **HR** | **95%CI** | **Z** | ***P* value** |  | **HR** | **95%CI** | **Z** | ***P* value** |  | **HR** | **95%CI** | **Z** | ***P* value** |
| **LINC00839** | 1.47 | 1.33-1.62 | 7.49 | < 0.001 |  | 1.22 | 1.13-1.31 | 5.25 | < 0.001 |  | 1.43 | 1.22-1.68 | 4.4 | < 0.001 |  | 1.29 | 1.16-1.44 | 4.71 | < 0.001 |
| **DUXAP8** | 1.47 | 1.33-1.62 | 7.71 | < 0.001 |  | 1.26 | 1.17-1.35 | 6.28 | < 0.001 |  | 1.49 | 1.24-1.79 | 4.3 | < 0.001 |  | 1.38 | 1.23-1.56 | 5.23 | < 0.001 |
| **FIRRE** | 1.48 | 1.31-1.68 | 6.3 | < 0.001 |  | 1.31 | 1.20-1.42 | 6.07 | < 0.001 |  | 1.41 | 1.16-1.72 | 3.43 | 0.001 |  | 1.40 | 1.22-1.59 | 4.96 | < 0.001 |
| **DSCR8** | 1.22 | 1.14-1.29 | 6.3 | < 0.001 |  | 1.12 | 1.07-1.18 | 4.41 | < 0.001 |  | 1.21 | 1.11-1.32 | 4.36 | < 0.001 |  | 1.20 | 1.12-1.27 | 5.62 | < 0.001 |
| **LINC01011** | 0.49 | 0.41-0.58 | -7.95 | < 0.001 |  | 0.62 | 0.54-0.71 | -7.15 | < 0.001 |  | 0.40 | 0.27-0.61 | -4.37 | < 0.001 |  | 0.60 | 0.45-0.82 | -3.27 | 0.001 |
| **TSC22D1-AS1** | 0.45 | 0.38-0.55 | -8.34 | < 0.001 |  | 0.59 | 0.51-0.67 | -7.49 | < 0.001 |  | 0.44 | 0.30-0.64 | -4.21 | < 0.001 |  | 0.57 | 0.44-0.76 | -3.97 | < 0.001 |
| **TPT1-AS1** | 0.40 | 0.34-0.48 | -9.74 | < 0.001 |  | 0.53 | 0.46-0.61 | -8.91 | < 0.001 |  | 0.28 | 0.18-0.45 | -5.31 | < 0.001 |  | 0.34 | 0.25-0.48 | -6.35 | < 0.001 |
| **LINC02381** | 0.68 | 0.62-0.76 | -7.37 | < 0.001 |  | 0.78 | 0.72-0.85 | -5.94 | < 0.001 |  | 0.65 | 0.54-0.78 | -4.56 | < 0.001 |  | 0.69 | 0.60-0.80 | -5.02 | < 0.001 |
| **AGPAT4-IT1** | 0.49 | 0.42-0.58 | -8.91 | < 0.001 |  | 0.62 | 0.55-0.70 | -7.68 | < 0.001 |  | 0.44 | 0.31-0.61 | -4.87 | < 0.001 |  | 0.54 | 0.42-0.69 | -4.96 | < 0.001 |
| **LOC100507557** | 0.57 | 0.50-0.65 | -8.37 | < 0.001 |  | 0.69 | 0.62-0.76 | -7.33 | < 0.001 |  | 0.56 | 0.46-0.68 | -5.69 | < 0.001 |  | 0.71 | 0.61-0.82 | -4.66 | < 0.001 |
| **CASC15** | 0.56 | 0.49-0.64 | -9.01 | < 0.001 |  | 0.67 | 0.61-0.74 | -7.97 | < 0.001 |  | 0.55 | 0.41-0.74 | -3.89 | < 0.001 |  | 0.55 | 0.45-0.67 | -5.74 | < 0.001 |
| **EPB41L4A-DT** | 0.59 | 0.53-0.65 | -9.76 | < 0.001 |  | 0.72 | 0.66-0.78 | -7.69 | < 0.001 |  | 0.55 | 0.42-0.71 | -4.52 | < 0.001 |  | 0.66 | 0.55-0.79 | -4.58 | < 0.001 |
| **LOC283177** | 0.46 | 0.39-0.53 | -10.11 | < 0.001 |  | 0.60 | 0.53-0.67 | -8.9 | < 0.001 |  | 0.50 | 0.35-0.72 | -3.78 | < 0.001 |  | 0.57 | 0.45-0.73 | -4.53 | < 0.001 |
| **LINC02145** | 0.61 | 0.54-0.69 | -8.18 | < 0.001 |  | 0.71 | 0.65-0.78 | -7.45 | < 0.001 |  | 0.58 | 0.43-0.76 | -3.84 | < 0.001 |  | 0.59 | 0.48-0.71 | -5.36 | < 0.001 |
| **ELOVL2-AS1** | 0.64 | 0.57-0.72 | -7.12 | < 0.001 |  | 0.76 | 0.69-0.83 | -5.75 | < 0.001 |  | 0.58 | 0.45-0.75 | -4.16 | < 0.001 |  | 0.65 | 0.55-0.77 | -4.92 | < 0.001 |
| **MIAT** | 0.49 | 0.42-0.56 | -9.91 | < 0.001 |  | 0.58 | 0.51-0.65 | -9.41 | < 0.001 |  | 0.65 | 0.54-0.77 | -4.72 | < 0.001 |  | 0.66 | 0.57-0.76 | -5.95 | < 0.001 |
| **FAM13A-AS1** | 0.49 | 0.43-0.56 | -10.72 | < 0.001 |  | 0.63 | 0.57-0.70 | -8.73 | < 0.001 |  | 0.68 | 0.57-0.82 | -4.06 | < 0.001 |  | 0.72 | 0.63-0.82 | -4.8 | < 0.001 |
| **EPHA5-AS1** | 0.66 | 0.60-0.72 | -9.32 | < 0.001 |  | 0.76 | 0.71-0.81 | -7.81 | < 0.001 |  | 0.67 | 0.58-0.79 | -4.97 | < 0.001 |  | 0.78 | 0.70-0.87 | -4.47 | < 0.001 |
| **LINC01138** | 0.65 | 0.60-0.71 | -9.91 | < 0.001 |  | 0.75 | 0.70-0.80 | -8.31 | < 0.001 |  | 0.59 | 0.48-0.72 | -5.17 | < 0.001 |  | 0.68 | 0.59-0.79 | -5.25 | < 0.001 |
| **LOC101928100** | 0.55 | 0.47-0.63 | -8.39 | < 0.001 |  | 0.68 | 0.62-0.75 | -8.22 | < 0.001 |  | 0.66 | 0.52-0.84 | -3.42 | 0.001 |  | 0.65 | 0.55-0.76 | -5.31 | < 0.001 |

**Table S2.** Multivariate Cox proportional hazards model survival analysis of the identified four lncRNAs in cohort 1.

| **LncRNA** | **Overall survival** | | | | |  | **Event-free survival** | | | | |
| --- | --- | --- | --- | --- | --- | --- | --- | --- | --- | --- | --- |
|  | **HR** | **95%CI** | **Z score** | **Coefficient** | ***P* value** |  | **HR** | **95%CI** | **Z score** | **Coefficient** | ***P* value** |
| **LINC00839** | 1.255 | 1.137-1.384 | 4.520 | 0.227 | < 0.001 |  | 1.099 | 1.024-1.181 | 2.600 | 0.095 | 0.009 |
| **FIRRE** | 1.318 | 1.147-1.514 | 3.900 | 0.276 | < 0.001 |  | 1.219 | 1.103-1.328 | 4.030 | 0.191 | < 0.001 |
| **LOC283177** | 0.593 | 0.486-0.724 | -5.140 | -0.522 | < 0.001 |  | 0.727 | 0.628-0.841 | -4.280 | -0.319 | < 0.001 |
| **LOC101928100** | 0.711 | 0.602-0.839 | -4.030 | -0.341 | < 0.001 |  | 0.806 | 0.721-0.902 | -3.750 | -0.215 | < 0.001 |

**Table S3.** Patients’ characteristics and multivariate Cox survival analysis of the four-lncRNA signature after adjusting for the clinical risk factors in the cohort 1.

|  |  | **No.** | **No. of**  **Death** | **No. of**  **Event** | **Overall survival** | | |  | **Event-free survival** | | |
| --- | --- | --- | --- | --- | --- | --- | --- | --- | --- | --- | --- |
|  |  |  |  |  | **HR** | 95%CI | ***P* value** |  | **HR** | **95%CI** | ***P* value** |
| **Age** | **< 18 m** | 300 | 20 | 68 |  |  |  |  |  |  |  |
|  | **≥ 18 m** | 198 | 85 | 115 | 1.415 | 0.789-2.536 | 0.244 |  | 0.979 | 0.646-1.483 | 0.920 |
| **MYCN amp** | **No** | 401 | 53 | 120 |  |  |  |  |  |  |  |
|  | **Yes** | 92 | 51 | 60 | 1.983 | 1.256-3.131 | 0.003 |  | 1.075 | 0.718-1.610 | 0.724 |
| **Risk** | **Low** | 322 | 13 | 63 |  |  |  |  |  |  |  |
|  | **High** | 176 | 92 | 120 | 3.237 | 1.362-7.695 | 0.008 |  | 2.214 | 1.210-4.049 | 0.010 |
| **INSS** | **1** | 121 | 1 | 9 |  |  |  |  |  |  |  |
|  | **2** | 78 | 4 | 19 |  |  |  |  |  |  |  |
|  | **3** | 63 | 14 | 27 |  |  |  |  |  |  |  |
|  | **4** | 183 | 82 | 115 | 1.337 | 0.756-2.366 | 0.319 |  | 1.146 | 0.741-1.772 | 0.541 |
|  | **4S** | 53 | 4 | 13 |  |  |  |  |  |  |  |
| **LncRNA score** | **Low** | 257 | 4 | 36 |  |  |  |  |  |  |  |
|  | **High** | 241 | 101 | 147 | 8.781 | 2.818-27.364 | < 0.001 |  | 3.279 | 2.010-5.349 | < 0.001 |

**Table S4.** Patients’ characteristics and multivariate Cox survival analysis of the four-lncRNA signature after adjusting for the clinical risk factors in cohort 2.

|  |  | **No.** | **No. of**  **Death** | **No. of**  **Event** | **Overall survival** | | |  | **Event-free survival** | | |
| --- | --- | --- | --- | --- | --- | --- | --- | --- | --- | --- | --- |
|  |  |  |  |  | **HR** | 95%CI | ***P* value** |  | **HR** | **95%CI** | ***P* value** |
| **Age** | **< 18 m** | 103 | 3 | 16 |  |  |  |  |  |  |  |
|  | **≥ 18 m** | 120 | 39 | 73 | 7.748 | 2.303-26.074 | 0.001 |  | 3.620 | 2.036-6.436 | < 0.001 |
| **MYCN amp** | **No** | 176 | 24 | 63 |  |  |  |  |  |  |  |
|  | **Yes** | 46 | 18 | 26 | 1.593 | 0.760-3.337 | 0.218 |  | 0.959 | 0.569-1.615 | 0.874 |
| **INSS** | **1** | 29 | 1 | 4 |  |  |  |  |  |  |  |
|  | **2** | 39 | 4 | 13 |  |  |  |  |  |  |  |
|  | **3** | 36 | 7 | 15 |  |  |  |  |  |  |  |
|  | **4** | 89 | 29 | 53 | 1.690 | 0.828-3.448 | 0.149 |  | 1.473 | 0.929-2.336 | 0.100 |
|  | **4S** | 30 | 1 | 4 |  |  |  |  |  |  |  |
| **LncRNA score** | **Low** | 152 | 14 | 40 |  |  |  |  |  |  |  |
|  | **High** | 71 | 28 | 49 | 2.311 | 1.085-4.923 | 0.030 |  | 2.591 | 1.580-4.251 | < 0.001 |
